# Linoleic acid induces metabolic stress in the intestinal microorganism *Bifidobacterium breve* DSM 20213

**DOI:** 10.1038/s41598-020-62897-w

**Published:** 2020-04-07

**Authors:** Alice Senizza, Gabriele Rocchetti, Maria Luisa Callegari, Luigi Lucini, Lorenzo Morelli

**Affiliations:** 1 Department for Sustainable Food Process, Università Cattolica del Sacro Cuore, via Emilia Parmense 84, 29122 Piacenza, Italy; 2 Centre for Research on Biotechnology (CRB), Università Cattolica del Sacro Cuore, via Milano 24, 26100 Cremona, Italy

**Keywords:** Biochemistry, Biological techniques, Chemical biology, Microbiology

## Abstract

Despite clinical and research interest in the health implications of the conjugation of linoleic acid (LA) by bifidobacteria, the detailed metabolic pathway and physiological reasons underlying the process remain unclear. This research aimed to investigate, at the molecular level, how LA affects the metabolism of *Bifidobacterium breve* DSM 20213 as a model for the well-known LA conjugation phenotype of this species. The mechanisms involved and the meaning of the metabolic changes caused by LA to *B. breve* DSM 20213 are unclear due to the lack of comprehensive information regarding the responses of *B. breve* DSM 20213 under different environmental conditions. Therefore, for the first time, an untargeted metabolomics-based approach was used to depict the main changes in the metabolic profiles of *B. breve* DSM 20213. Both supervised and unsupervised statistical methods applied to the untargeted metabolomic data allowed confirming the metabolic changes of *B. breve* DSM 20213 when exposed to LA. In particular, alterations to the amino-acid, carbohydrate and fatty-acid biosynthetic pathways were observed at the stationary phase of growth curve. Among others, significant up-regulation trends were detected for aromatic (such as tyrosine and tryptophan) and sulfur amino acids (i.e., methionine and cysteine). Besides confirming the conjugation of LA, metabolomics suggested a metabolic reprogramming during the whole growth curve and an imbalance in redox status following LA exposure. Such redox stress resulted in the down-accumulation of peroxide scavengers such as low-molecular-weight thiols (glutathione- and mycothiol-related compounds) and ascorbate precursors, together with the up-accumulation of oxidized (hydroxy- and epoxy-derivatives) forms of fatty acids. Consistently, growth was reduced and the levels of the oxidative stress marker malondialdehyde were higher in LA-exposed *B. breve* DSM 20213 than in the control.

## Introduction

Linoleic acid (LA), or 9-cis, 12-cis-octadecadienoate, is a polyunsaturated omega-6 fatty acid that typically occurs in nature as a triglyceride ester. LA has long attracted the attention of nutritionists, particularly for its main isomers, also known as conjugated linoleic acid (CLA). Diet is the only way to obtain the proper ratio of essential fatty acids and, according to current regulations, infant formula must contain LA and α-linolenic acid (ALA)^1^.

The role of LA in bacterial growth is not completely clear, even though different hypotheses have been proposed. Among these hypotheses is that LA is toxic to many bacteria, given its ability to reduce bacterial growth. Koppová and colleagues^2^ reported that the length of the lag phase was dependent on LA concentrations and was proportional to the fatty acid concentration. In particular, long-chain fatty acids with a higher number of unsaturated double bonds have a major inhibitory effect on bacterial cell growth compared to fatty acids with fewer unsaturated bonds. Bacteria adopt many strategies to survive in the presence of LA, such as hydrogenating unsaturated free fatty acids into more saturated products, which are considered to be less toxic^3^. Bifidobacteria have been deeply studied for their ability to transform LA into different CLA isomers, including 9-cis, 11-trans-octadecadienoate (9-cis, 11-trans-CLA) and vaccenic acid^4^. In previous works, several research groups have evaluated the ability of different bifidobacteria to convert LA into CLA-isomers. Raimondi *et al*.^4^ screened 34 bifidobacteria strains and reported that *B. breve* WC0421 was the best CLA producer, converting LA into 68% 9-cis, 11-trans-CLA and 25% 9-trans, 11-trans-CLA. Additionally, O’Connell *et al*.^5^ used gas chromatography (GLC) to estimate the CLA-production capabilities of different *Bifidobacterium* species, showing that *B. breve* NCFB 2258 was the strain providing the best LA transformation to CLA (namely to 9-cis, 11-trans-CLA). Such positional and geometric conjugated isomers of the essential fatty-acid LA have received great attention from the scientific community because they have been linked to many health-promoting activities, such as anti-adipogenic, anti-diabetogenic and anti-atherosclerotic activities^6^. Nonetheless, the conjugation of LA to CLA has been reported among the gut commensal-produced processes involved in host-microbe interactions^7^.

Despite such important outcomes, the mechanism(s) of bifidobacteria for metabolizing LA is still unclear and information about the bacterial toxicity of this compound remains limited^8^. In fact, to the best of our knowledge, no significant information regarding the comprehensive changes induced by LA to *B. breve* metabolism has been reported. In this regard, targeted/untargeted metabolomics platforms could offer a powerful tool for discovering novel compounds and biomarkers that result from modifications induced by different treatments^9^. Both previously cited metabolomics-based strategies are characterized by inherent advantages and disadvantages. In particular, untargeted metabolomics represents the comprehensive analysis of all the measurable analytes in a sample, including chemical unknowns^9^. Due to its comprehensive nature, untargeted metabolomics must be coupled with advanced chemometric techniques, such as multivariate analysis, to reduce the extensive datasets generated by the smaller set of manageable signals. In such a way, this untargeted strategy can help to better understand the complexity behind the metabolic changes of bacteria exposed to exogenous factors^9^. In fact, when predefined lists of analytes are studied (targeted metabolomics), it is more difficult to find correlations between sets of metabolites and specific physiological states^10^.

Therefore, the aim of the present work was to apply an untargeted metabolomics approach using liquid chromatography time-of-flight mass spectrometry (UPLC-ESI-QTOF-MS) with multivariate statistical analysis to study the metabolomic changes in *B. breve* DSM 20213 exposed to LA.

## Results and Discussion

### Growth and metabolomic profile of *B. breve* DSM 20213 following exposure to LA

**Figure 1:**
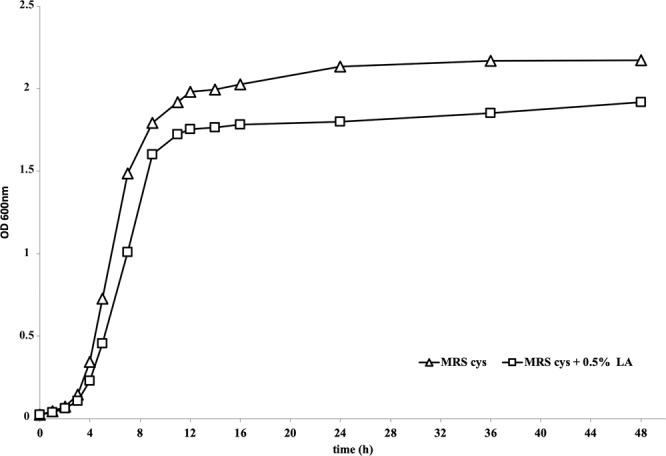
Growth curve of *B. breve* DSM 20213 cultivated for 48 h in MRS Cys either with or without addition of linoleic acid (LA). LA was added to MRS medium at the final concentration of 0.5 g/L.

Under our experimental conditions, the addition of LA to the medium negatively affected the growth rate of *B. breve* DSM 20213. In fact, the cell number of the strain grown in MRS + LA was lower than in MRS, starting from the earlier points of the growth curve (Fig. 1), thus demonstrating a reduced growth rate compared to MRS throughout the whole curve. After 48 h culture, the cell number of the strain grown in MRS + LA had an FC value = 0.89, hence exhibiting a reduced growth rate (−11%) compared to MRS. Consistently, the generation time was lower in LA-treated cells than in control, both in the log (50 min *vs* 44 min) and in the stationary phase (296 min *vs* 120 min).

In order to assess the major metabolic changes promoted by LA, a UHPLC-QTOF mass spectrometric method was used to depict the metabolism of *B. breve* DSM 20213, as elicited by the addition of LA to the medium. In this regard, the untargeted approach resulted in the putative annotation of more than 10,000 features using the comprehensive database MetaCyc^11^ (www.metacyc.org). Supplementary File 1 presents the detailed list of annotated metabolites and composite mass spectra (mass & abundance combinations).

**Figure 2:**
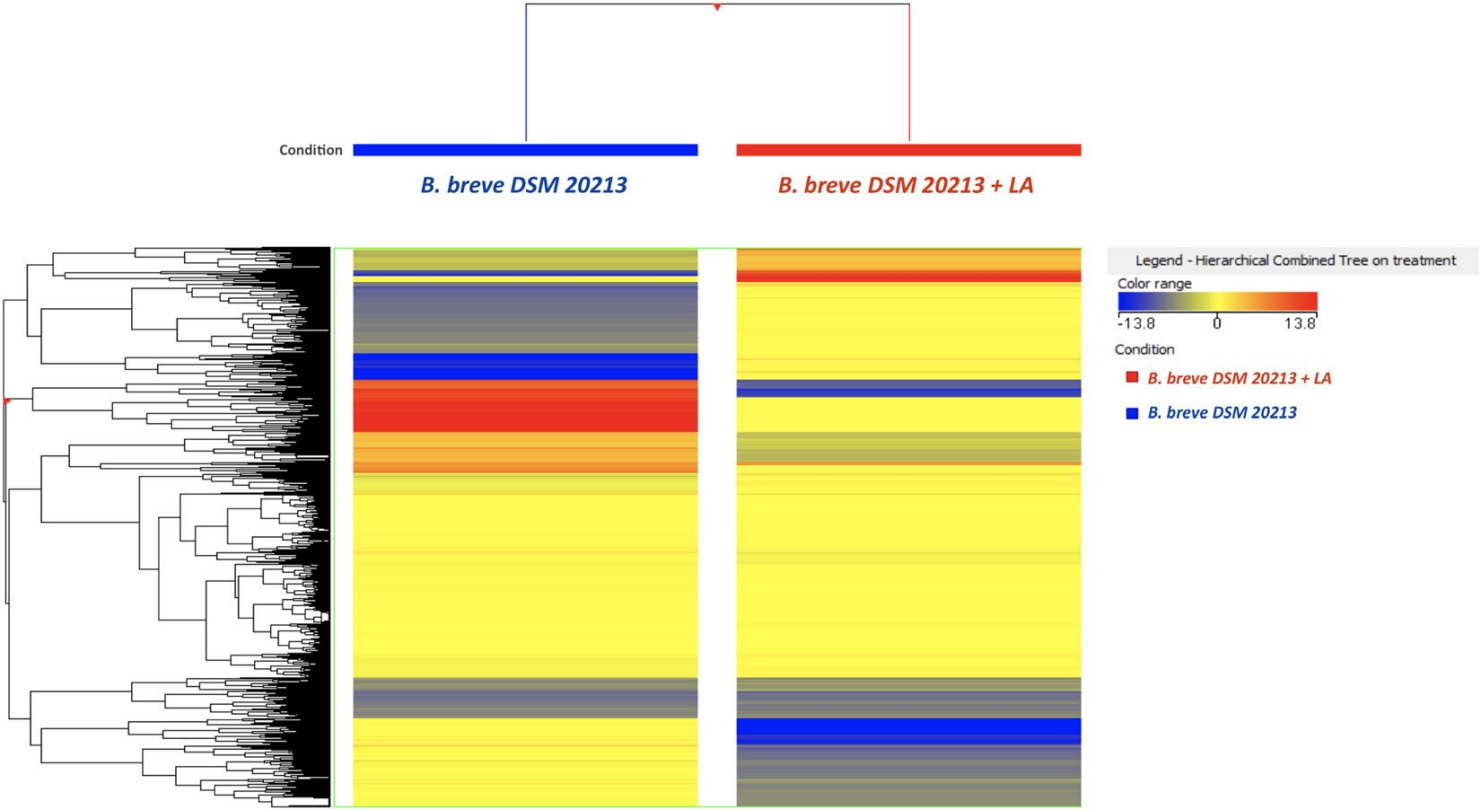
Unsupervised hierarchical cluster analysis (HCA) of the metabolite profile of *B. breve* DSM 20213 + linoleic acid (LA) *vs B. breve* DSM 20213 (similarity: ‘Euclidean’; linkage rule: ‘Ward’) following 48 h of exposure. Compound intensity was used to build up the heat map, on the basis of which the clusters were generated. This figure was generated in Mass Profiler professional version B.12.04 (Agilent technologies).

Overall, the large number of metabolites detected reflects the complexity of the mechanism under investigation and increased the chances of depicting the metabolic changes induced by the addition of LA. Indeed, the analysis of complex matrices such as microbial samples comprises a huge amount of metabolites with high chemical diversity, thus requiring an untargeted metabolomics approaches to profile all the unknowns^12^. In such metabolomics-based studies, post-acquisition steps (i.e., feature extraction, mass and retention time alignment, filtration and normalization) are required before applying multivariate analysis. In our experiments, this process reduced the number of features but improved confidence in the annotation. Thereafter, in order to represent similarities/dissimilarities in metabolic profiles between the two sample groups (i.e., *B. breve* DSM 20213 and *B. breve* DSM 20213 + LA) at the end of the growth curve, an unsupervised hierarchical cluster analysis (HCA) was carried out using the Mass Profiler Professional software (Agilent Technologies). The output of this analysis is reported in Fig. 2 as the average value of the three replicates. The clustering produced from the heat map based on Fold-Change distribution clearly shows the effect of adding LA on the metabolism of *B. breve* DSM 20213; in particular, it is evident that LA induced a clear reduction in specific groups of compounds and promoted the accumulation of other metabolites.

**Figure 3:**
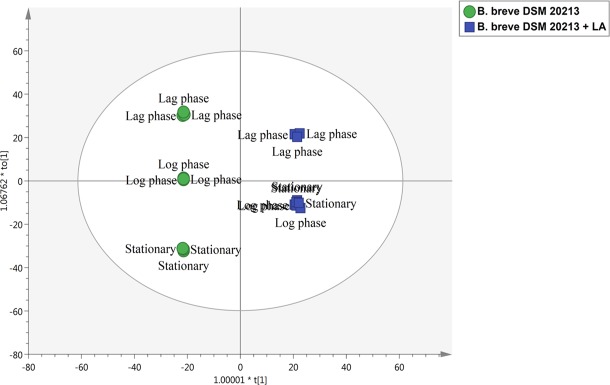
Supervised Orthogonal Projections to Latent Structures Discriminant Analysis (OPLS-DA) score plot on the metabolite profile of *B. breve* DSM 20213 + linoleic acid (LA) *vs. B. breve* DSM 20213. Samples were collected at the end of lag phase, during the log phase and at the stationary phase; individual replications (*n* = 3) are given in the class prediction model score plot. This figure was produced in Simca 13 (Umetrics).

Multivariate analysis supervised methods, such as partial least squares and orthogonal projection to latent structures discriminant analysis (i.e., PLS-DA and OPLS-DA, respectively), are typically used following unsupervised tools, such as HCA, to better identify discriminant compounds^13^. In fact, although unsupervised cluster analysis can reveal differences between classes without supervision (based on intrinsic similarities in their measurements), the utilization of a class membership characterizing supervised models allows better separation between classes in the score space. Additionally, the Y-predictive variability is better separated from the Y-uncorrelated in X (i.e., orthogonal signal correction) when OPLS-DA is applied. This analysis was carried out including the samples collected at the end of the lag phase, during the log phase and at the stationary phase (Fig. 3). Interestingly, although distinct metabolomic profiles could be found at the different points of the growth curve within each treatment, two distinct clusters could be identified; these clusters included samples from bacteria grown in LA and without LA, respectively. However, although three distinct sub-clusters could be observed in cells grown without LA, the samples gained from *B. breve* DSM 20213 following exposure to LA and gained from exponential and stationary phase presented overlapping metabolomic profiles. These patterns suggest that the effect of LA addition imposed a metabolic reprogramming in *B. breve* that could be observed irrespective of the growth phase, from the exponential up to the stationary phase. Indeed, the OPLS-DA score plot (Fig. 3) indicates that LA-related metabolomic changes were comparable between exponential and stationary phases in LA-treated cells. On the contrary, three distinct clusters could be observed in control, each of them corresponding to a different growth stage (namely lag, log and stationary phases). The different patterns observed for control and LA-treated cells suggest that LA imposed a metabolic reprogramming that was hierarchically prevalent irrespective of the growth phase considered and particularly evident starting from the log phase (where the samples overlapped - Fig. 3). According to our results, the separation of treatments in the OPLS-DA hyperspace was effective, as suggested by model’s validation parameters (R^2^Y = 0.99 and Q^2^Y = 0.99). Furthermore, adequate cross-validation parameters and permutation test outputs were obtained, thus confirming the robustness of the multivariate models. Despite several information related to lag phase are herein provided, it is important to keep in mind that several confounding factors may affect the results. Since the bacterial population in this phase is represented by an overnight culture inoculum, it is reasonable that cells are not synchronized. Thus, the metabolites found in this phase could be released by the dead, damaged and viable cells that constituted the inoculum population.

A VIP analysis, followed by fold-change (FC) analysis, was carried out to identify the best discriminants from both the following OPLS-DA modelling. By running the relevant features (i.e., FC > 2 and VIP score > 0.8) in the Pathway Tools software, the metabolic perturbations induced by LA in *B. breve* DSM 20213 were computationally predicted. Table 1 reports the main biosynthetic pathways highlighted at each sampling point, together with the average and cumulative FC values. The cumulative fold change of the metabolisms affected by LA addition is also graphically reported as Supplementary File 2.

**Table 1:** Biosynthetic pathways considering B. breve DSM 20213 + linoleic acid (LA) vs B. breve DSM 20213, as resulted by Pathway Tools Omics Dashboard for MetaCyc (www.metacyc.org).

		*B. breve* DSM 20213 + LA *vs B. breve* DSM 20213 (lag phase)	*B. breve* DSM 20213 + LA *vs B. breve* DSM 20213 (log phase)	*B. breve* DSM 20213 + LA *vs B. breve* DSM 20213 (stationary phase-48h)
Amino acids	LogFC (average)	4	−7	−1
	LogFC (cumulative)	34	−52	−17
Fatty Acids and Lipids	LogFC (average)	8	−7	0.8
	LogFC (cumulative)	291	−259	31
Carbohydrates	LogFC (average)	5	−12	−0.3
	LogFC (cumulative)	31	−72	3
Amines and polyamines	LogFC (average)	5	−11	7.3
	LogFC (cumulative)	33	−74	44
Cofactors	LogFC (average)	4	−7	−0.4
	LogFC (cumulative)	94	−158	−21
Nucleosides-Nucleotides	LogFC (average)	10	−7	−6.2
	LogFC (cumulative)	62	−39	−56
Secondary metabolites	LogFC (average)	0	−10	0.3
	LogFC (cumulative)	−40	−1422	135
Cell structures	LogFC (average)	11	−6	1.2
	LogFC (cumulative)	112	−62	10
Metabolic regulators	LogFC (average)	10	−10	−0.4
	LogFC (cumulative)	50	−48	−3
Other biosynthesis	LogFC (average)	7	−7	−1.8
	LogFC (cumulative)	66	−70	−41

Each main pathway is provided together with the average Log Fold Change value (LogFC) and the cumulative LogFC value, considering lag, log and stationary (48 h) phases.

As it can be noted differences in metabolic profiles started to be visible at the end of the lag phase, to become marked during the log phase. In this latter stage, a general down accumulation could be evidenced in LA treated cells, regardless the biochemical class considered. This suggests that the microorganism was facing a stress condition that impaired its metabolic capability, in agreement with both the reduced growth rate we observed from the end of the lag phase (Fig. 1) and the higher generation time observed in LA-treated cells. Senizza and co-authors^14^ observed a similar growth reduction, when the same strain, DSM 20213, was exposed to LA. We can therefore speculate that the increased demand to allocate cell resources towards the coping of stress-induced processes resulted in such reduction of growth rate following addition of LA.

The VIP variables selection method was used to rank the contribution of each variable for discrimination purposes, i.e., to highlight the best markers of the OPLS-DA distribution. In particular, the VIP selection method allowed reducing the high complexity of the metabolomics-based dataset and listed 1,171 metabolites (Supplementary File 1). As provided, 1672 metabolites were characterized by a VIP score > 1 (extremely important in the model), while the remaining 895 metabolites showed VIP scores between 0.8 and 1 (moderately important in the model). Fold-change values (FC) were produced for discriminant compounds and, finally, this list of compounds was exported into the Pathway Tools Omics Dashboard^15^ for interpretation.

### Changes induced by LA to primary metabolism and membrane lipids

The rise of ‘omics’ technologies (such as high-resolution mass spectrometry combined with different chemometric tools) resulted in a huge influx of complex, high-resolution datasets, thus complicating the understanding of these data from a biological point of view^15^. Therefore, to reduce the complexity of the metabolomics-based data, the OPLS-DA VIP discriminant markers were loaded into the Pathway Tools Omics Dashboard. By running the relevant features (i.e., FC > 2 and VIP score > 0.8) in the Pathway Tools software, the metabolic perturbations induced by LA in *B. breve* strain were computationally predicted. Table 1 reports the main biosynthetic pathways highlighted, together with the number of discriminant compounds and the average and cumulative FC values throughout the different stages of the bacterial growth curve.

Among others, the primary metabolism involving amino acids, fatty acids and carbohydrates was affected by LA addition. Comparable results were observed in *B. breve* DSM 20213 exposed to LA, at transcriptome level^14^. These authors found that LA impacted the expression of genes involved in carbohydrate and amino acids transport. In particular, a negative regulation of genes involved in sugar transport, such as the PTS system and permease proteins of ABC transporters rather than oligopeptide permeases. Furthermore, they observed a down regulation of a type I multifunctional fatty acid synthase.

As can be observed, secondary metabolites and cofactors were also modulated by LA. Nonetheless, several compounds in these classes are involved in more than one pathway, thus complicating the specific understanding of their metabolic roles^16^. However, these alterations suggest that the metabolic reprogramming induced by LA was rather wide and also included secondary metabolism processes, some of which are described later.

The ability of *B. breve* to convert LA into different CLA-isomers has been deeply studied^8^, with 9-cis, 11-trans-CLA recognized as the most predominant isomer produced. Consistently, our metabolomics approach allowed putatively annotating different CLA isomers (such as 10-trans, 12cis-CLA and 9-cis, 11-trans-CLA). However, as reported in Supplementary File 1, the principal CLA isomer (9-cis, 11-trans-CLA) was putatively annotated only in *B. breve* DSM 20213 grown in LA-containing medium to record LogFC values of 3.5 and 15.9 at log and stationary phases, respectively. Overall, the response of *B. breve* to the presence of LA is a multifactorial process that involves many mechanisms and cellular pathways^17^.

The most discriminant metabolites highlighted by VIP analysis following OPLS-DA are provided both in Table 2 (*B. breve* DSM 20213 + LA *vs B. breve* DSM 20213, 48 h of exposure) and supporting material (Supplementary File 3). Surprisingly, in our experimental conditions, the addition of LA to the medium showed a dramatic influence on amino acid metabolism. In particular, a strong up-regulation trend was found for the two sulfur amino acids, L-methionine and L-cysteine, in *B. breve* DSM 20213 + LA *vs B. breve* DSM 20213 (LogFC = 7.0 and 13.0, respectively). Interestingly, L-cysteine was found to possess one of the highest VIP scores following OPLS-DA analysis (Table 2), thus confirming its importance for predicting the impact of LA on *B. breve* DSM 20213. Additionally, we noticed a down-regulation trend for L-cystathionine (LogFC = −13.3), a putative intermediate of the previously cited amino acids pathway, as reported by Lee and O’Sullivan^18^ in bifidobacteria. Previous literature confirms that LA induces an up-regulation of cystathionine beta lyase *metC* in *B. breve* DSM 20213^14^.

**Table 2:** Discriminant compounds (as resulted by OPLS-DA) better discriminating the changes on primary metabolism and redox stress. Each compound is provided together with the corresponding VIP score, LogFC value (*B. breve* DSM 20213 + LA *vs B. breve* DSM 20213) and accumulation.

	VIP score (OPLS-DA)	LogFC (*B. breve* DSM 20213 + LA *vs B. breve* DSM 20213)	Accumulation
***Amino acids***			
L-Cystathionine	1.10 ± 1.17	−13.3	Down
L-Homocysteine	0.90 ± 1.19	−0.6	Down
L-Methionine	0.83 ± 1.09	7.0	Up
L-Cysteine	1.45 ± 0.19	13.0	Up
4-hydroxyphenylpyruvate	1.15 ± 1.13	−13.4	Down
Shikimate 3-phosphate	1.22 ± 1.05	−11.5	Down
1-(*o*-carboxyphenylamino)-1′-deoxyribulose 5′-phosphate	1.45 ± 0.09	−18.6	Down
L-tryptophan	1.30 ± 0.78	12.4	Up
L-tyrosine	1.14 ± 0.79	15.0	Up
Imidazole-lactate	1.15 ± 0.87	−14.0	Down
L-histidine	0.94 ± 1.50	7.7	Up
***Fatty acids***			
9-cis, 11-trans-octadecadienoate	0.95 ± 1.17	15.9	Up
Vernolic acid	1.01 ± 1.23	8.3	Up
2-hydroxytricosanoate	1.05 ± 1.36	1.6	Up
2-hydroxydocosanoate	1.04 ± 1.08	1.3	Up
4-oxopentanoate	0.86 ± 0.61	12.8	Up
Auricolate	1.12 ± 1.41	1.7	Up
16-hydroxy-15-methyl-palmitate	1.21 ± 0.73	17.8	Up
***Stress-related compounds***			
L-sorbosone	1.15 ± 1.32	−14.9	Down
2-phospho-L-ascorbate	1.45 ± 1.04	−17.6	Down
Glutathione amide	0.89 ± 0.87	−8.2	Down
Glutathione amide disulfide	0.92 ± 0.89	−7.1	Down
Glutathione amide perthiol	1.00 ± 1.14	−11.8	Down
Mycothiol	1.41 ± 0.62	−17.9	Down
Mycothiol-bimane conjugate	1.15 ± 1.10	−13.4	Down
2-aminoprop-2-enoate	1.44 ± 1.12	−17.3	Down

Such up-accumulation trends recorded for the two sulfur amino acids could indicate that the addition of LA has a similar impact on acid pH, as reported by Sanchez *et al*.^19^, who studied the influence of acid pH conditions on sulfur amino acid metabolism in *Bifidobacterium longum*. In addition, Jin and co-authors^20^ suggested improving the acid-resistance of bifidobacteria by adding cysteine and cystathionine into the acid-stress medium. Consistently, cysteine has been reported to play a protective role against detrimental effects of LA in *B. breve* DSM 20213^14^. Unfortunately, the reason(s) for these changes remains unclear and further *ad-hoc* investigations seem necessary to confirm these hypotheses.

The earlier time points of the growth curve evidenced a down-accumulation of some amino acids. In more detail, the hydrophobic L-valine (LogFC −18) and L-leucine/L-isoleucine (LogFC −20) exhibited negative trends at the log phase, and the same trend was observed for the polar amino acid L-histidine (LogFC −18). Other significant changes following the addition of LA to the medium were observed for aromatic amino acids, particularly when considering the biosynthesis of L-tyrosine and L-tryptophan, at the end of the stationary phase. In fact, these two compounds showed LogFC values of 15.0 and 12.4 respectively, at the stationary phase, thus revealing clear up-accumulation trends (Table 2). These trends were confirmed by looking at some of the most important biosynthesis intermediates, such as 4-hydroxyphenylpyruvate (Log FC = −13.4) and 1-(2-carboxyphenylamino)-1-deoxy-D-ribulose 5-phosphate (Log FC = −18.6), which are characterized by clear down-accumulated values. Taken together, these findings suggest that the addition of LA to the medium might result in the activation of the shikimate pathway that is responsible for the metabolism of aromatic amino acids^21^. Indeed, aromatic amino acids could be associated with a stress condition response, as observed by An and co-authors^22^, who analyzed bile-stress responses in *B. longum* BBMN68. Moreover, hydrophobic amino acids, such as aromatic amino acids, could protect proteins against stress mechanisms by building, for example, hydrophobic areas^22^. Finally, another up-regulation trend was observed at 48 h for L-histidine (Log FC = 7.7), likely arising from the degradation of imidazole-lactate, which was found to possess strong down-regulation (Log FC = −14.0).

Unlike for amino acids, the addition of LA did not cause marked changes in carbohydrate metabolism. Differences in carbohydrates accumulation were negligible at the earlier points of the growth curve. Thereafter, as shown in Table 1, the carbohydrate biosynthetic pathway is characterized by an average FC value = −0.3 at 48 h of exposure. Polysaccharides are believed to contrast cell aggregation and adhesion^23^, suggesting that a decrease of these compounds could be a strategy of *B. breve* DSM 20213 to improve the hydrophobicity and auto-aggregation of its cells when exposed to LA. Previously, Shakirova *et al*.^24^ reported an increase of cell surface hydrophobicity in relation to decreased carbohydrate levels in *Bifidobacterium lactis* Bb12.

Regarding fatty acids, a significant impact of LA on fatty acid (FA) and membrane lipids profile was observed throughout the whole growth curve (Table 1 and Supplementary File 3). In detail, glycolipids and phospholipids with a high degree of unsaturation underwent a complex modulation, with a tendency to up-accumulate at the lag phase, followed by down-accumulation starting from the exponential phase (Supplementary File 3). Interestingly, different papers described that the modification of membrane composition is among the primary effects imposed by LA on bacterial cells^14,25^. These authors reported that, among others, myristic, stearic and lactobacillic acids decreased following LA addiction. In our experiments, we could observe a strong decrease of stearic acid (LogFC = −1.0 and −19.0 at the lag and exponential phase, respectively). These changes in membrane lipids profile reflect the adaptation of our strain to LA, provided that modification of membrane composition plays a pivotal role in membrane fluidity, bilayer thickness and membrane-related functions^26^. Nonetheless, it is known that alteration of cell membrane is a mechanism often adopted by bacteria to cope with stress conditions^27^.

Regarding FA, we also highlighted an up-accumulation trend for some hydroxy- and oxo-fatty acids, such as 18-oxo-oleate (LogFC = 19.0 and 2.0 at the lag and exponential phase, respectively), 18-hydroxy-oleate (LogFC = 22.0 and 3.0 at the lag and exponential phase, respectively) and 18-hydroxy-stearate (LogFC = 19.0 and 1.0 at the lag and exponential phase, respectively). At the late stationary phase (48 h), 2-hydroxytricosanoate (LogFC = 1.6), 2-hydroxydocosanoate (LogFC = 1.3), 4-oxopentanoate (LogFC = 12.8), auriculate (LogFC = 1.7) and 16-hydroxy-15-methyl-palmitate (LogFC = 17.8) were also accumulated. Consistently, gut microbiota has been reported to produce hydroxy- and oxo-FAs from LA^28^. Additionally, Fernández-Murga and colleagues^29^ noticed in *Lactobacillus acidophilus* an increase in hydroxyl-fatty acids when this bacterium grows at suboptimal temperatures. In particular, they suggested a hypothetical role of these fatty acids in cell membrane permeability. In our experimental conditions, we found an increase of epoxides of LA, such as vernolic acid (Table 2 and Supplementary File 3). This latter compound had a VIP score > 1 and a LogFC accumulation of 19.0, 2.0 and 8.0 at the lag, log and stationary phases respectively, thus confirming the importance of this LA-derived metabolite in the OPLS-DA prediction model. Interestingly, in a previous work^30^, some authors reported an increase (up to 37% of total fatty acids) of vernolic acid in *Lactobacillus helveticus* as a response to salt, acid, oxidative and thermal stresses, thus outlining a possible correlation of this class of compounds with stress conditions. However, further studies (e.g., using targeted analytical approaches) are needed to confirm this trend.

### Effect of LA on redox stress

Besides the metabolic reprogramming induced by LA, several discriminant compounds could be related to redox stress and oxidative imbalance in *B. breve* DSM 20213. Indeed, the coordinated regulation of ubiquinol and menaquinol species starting from the earlier steps of the growth curve (Supplementary File 3), together with the changes in ascorbate, glutathione and mycothiol species observed at 48 h of LA exposure (Table 2), suggests an increased capacity of *B. breve* DSM 20213 to counteract intracellular radical species. At the end of the growth curve, 3-demethylubiquinol-7 and ubiquinol-6 presented LogFC values of 8.4 and 16.4 respectively, whereas demethylmenaquinol-7 and demethylmenaquinol-9 exhibited LogFC values of 2.9 and 2.1 respectively. The accumulation of membrane-related quinol/quinones such as menaquinols can be linked to the need to limit accumulation of superoxide and the production of H_2_O_2_^31^. Since bifidobacteria lack of respiratory chain, NAD^+^ is regenerated by lactate dehydrogenase and flavoproteins. However, when lactate is accumulated (i.e., during the stationary phase), these ways of NAD^+^ regeneration are replaced by NADH oxidase and/or peroxidase activities, a process that generates toxic H_2_O_2_^32,33^. More recently, the genome analysis of *B. breve* UCC2003^34^ revealed the presence of a putative bd-type quinol oxidase subunit II gene, coding for an oxidoreductase, present also on the *B. breve* DSM 20213 chromosome. This oxidase has been proposed to protect sensitive enzymes and cells from ROS^35^ and could represent one of the putative mechanisms used by bifidobacteria for limiting the intracellular accumulation of superoxide.

Consistently, a down-accumulation of L-sorbosone (ascorbate precursor, LogFC = −14.9), glutathione amide, its disulfide-related and perthiol derivatives (LogFC = −8.2, −7.1 and −11.8, respectively) as well as mycothiol and its bimane conjugate (LogFC = −17.9 and −13.4, respectively) were recorded at late stationary phase. These low molecular weight thiols normally increase during the late exponential and stationary phases to play an important role in redox balance of the cell^36^. We can therefore speculate that the modulation of these compounds represents the adaptive consequence of LA-mediated oxidative stress in *B. breve* strain. The glutathione-related metabolite 2-aminoprop-2-enoate was also altered in presence of LA. Glutathione and other low molecular weight thiols are redox buffers known to be involved, in bacteria, in peroxide detoxification processes and early adaptation to oxidative stress^37^. Interestingly, glutathione has also been linked to low pH and other environmental stress factors^38^. Such radical inactivation roles might also involve the thioredoxin system, which has been reported to regulate dithiol/disulfide balance in bacteria thanks to its disulfide reductase activity^39^. Further confirmation can be achieved from the reduced content of mycothiol and its derivative. This compound functions as a reserve of cysteine and in the detoxification of reactive oxygen and nitrogen species by acting as a thiol buffer protecting against disulfide stress^40^. Notably, Actinobacteria have been linked to a wide diversity of low-molecular-weight protective thiols to manage oxidative stress and environmental challenges^41^. Indeed, transcriptomic evidences and *ad-hoc* growth experiments have demonstrated the protective role of cysteine towards the LA-induced stress in *B. breve* DSM 20213^14^.

Another clue that relates LA exposure to oxidative imbalance in *B. breve* DSM 20213 is the trend observed for L-sorbosone that suggests a reduced production of ascorbate. Like the previously-mentioned thiols, ascorbate has a pivotal role in defending bacteria against oxidative imbalances and has been linked to cell growth^42,43^. Consistently with our postulation, Senizza *et al*.^14^ highlighted a strong up-regulation of the gene encoding for WhiB-like protein WblE in *B. breve* DSM 20213 exposed to LA. This protein is an iron-sulfur protein having a redox-sensing function, likely functioning as disulfide reductase^36^, whose gene results up regulated under stress conditions. Notably, Wbl proteins have been related to mycothiol and, together with cysteine and glutathione, they have been recognized to act as protective factors towards oxidative stress and to play a major role in thiols homeostasis by averting disulfide stress^36^.

Consistent with our postulation, the previously discussed up-accumulation of several hydroxy- and oxo-fatty acids throughout the whole growth curve can be related to a reduced capacity to cope with oxidative stress. The same can be observed by looking at MDA content. This compound, widely recognized as a marker of lipid peroxidation, was significantly higher (*p* < 0.05) in LA-treated *B. breve* cells when compared with control, recording positive fold-change values during the whole growth curve (p < 0.05, n = 3). In more detail, MDA ranged from not detectable values at the lag phase, to fold-change values of 8.2 (17.2 *vs* 2.1 nM) and 14.9 (19.4 *vs* 1.3 nM) at the log and stationary phases, respectively. Similar trends were reported by Senizza *et al*. in the same strain in presence of LA^14^. Therefore, it is possible to postulate an association between the lipid oxidation phenomena and the previously reported reduced growth rate. Indeed, He *et al*.^44^ reported that the ability to cope with oxidative stress is a key factor in dramatically improving cell survival in bifidobacteria.

Taken together, the clues related to the redox status of *B. breve* DSM 20213 following LA exposure suggest that the bacteria underwent a metabolic reprogramming that resulted in a modulation of the ability to contrast oxidative species. As a consequence, such bacteria experienced an imbalanced regulation of the redox status, which might be linked to the reduced growth we observed.

## Conclusions

In this work, untargeted metabolomics coupled with multivariate statistics were used to shed light on the impact of LA on *Bifidobacterium breve* DSM 20213 metabolism. To date, most research has focused only on the conjugating ability of bacteria and their correlations with the host’s health^45^. Overall, our findings corroborate the hypothesis that the addition of LA to the medium produces a stress/detoxification response. The growth of *B. breve* DSM 20213 was reduced following addition of LA. Metabolomics clearly highlighted distinctive metabolic signatures in this strain when the LA was added to the medium. In fact, *B. breve* DSM 20213 seems to adopt different strategies to survive the addition of LA, via changing its metabolism. However, the response of this strain to LA seems to be similar to those induced by other stress factors, such as those involving oxidative imbalance and low-molecular-weight thiols, membrane quinones and ascorbate. However, further and more focused work is still necessary to deepen the present results, *e.g*., by using other analytical and complementary approaches such as transcriptomic and/or targeted approaches.

## Methods

### Chemicals, strain and culture conditions

Linoleic and formic acids were purchased from Sigma-Aldrich (St. Louis, USA), while water and methanol (both LC-MS grade) were from VWR (Milan, Italy).

*B.breve* was obtained from the DSMZ collection (Braunzschweig, Germany). *B. breve* DSM 20213 was cultured in MRS broth (BD Difco™ Lactobacilli MRS broth, Fisher Scientific) that was modified by adding 0.5 g/L L-cysteine-HCl. In order to study the biotransformation of LA into CLA, 0.5 g/L of LA was added to MRS and the broth was then filter sterilized (0.22 µm)*. B. breve* DSM 20213 was cultured in triplicate in both MRS and MRS + LA at 37 °C for 48 h under anaerobic conditions. Growth curves were built by measuring optical density at 600 nm (OD600) every hour in the first 12 h, and then at 14, 16, 24, 36 and 48 h. The generation time was calculated using the number of cells enumerated at the lag phase (3 h for both MRS and MRS + LA), at the log phase (5 h and 6 h for MRS and MRS + LA, respectively) and at the beginning of stationary phase (7 h and 8 h for MRS and MRS + LA, respectively). Samples were taken at the end of lag phase, during the exponential phase and at the stationary phase (3, 5 and 48 h respectively); the cultures in MRS and MRS + LA were quenched in 80% methanol, centrifuged at 10,000 × *g* for 10 min and the pellets obtained were resuspended in 1 mL of sterilized nuclease-free water and immediately stored at −20 °C until analysis, while the supernatants were discarded. MRS agar plus 0.5 g/L L-cysteine-HCl was used for plate counts.

### Malondialdehyde (MDA) determination

The MDA content was measured at lag, log and stationary phases by using the thiobarbituric acid reactive substance (TBARS) assay, as previously reported^46^. Pellets were obtained from 10 mL of cell culture by centrifuging for 10 min at 10,000 × *g*. The absorbance at 532 nm was determined using a Perkin Elmer (Ontario, Canada) Lambda 12 spectrophotometer. For MDA determination, a molar extinction coefficient of 155 cm^−1^ mM^−1^ was used. Results were finally expressed as nM MDA equivalents (*n* = 3).

### UHPLC-ESI-QTOF untargeted profiling and data processing

The previously obtained resuspended cell aliquots were sonicated for 5 min, centrifuged at 10,000 × *g* for 10 min, and then supernatants were filtered using 0.22 μm cellulose syringe filters into amber vials until further analysis. The comprehensive metabolite profile of the extracts was then investigated using UHPLC-QTOF mass spectrometry. In particular, the instrumentation consisted of a 1290 UHPLC coupled with a G6550 quadrupole-time-of-flight (QTOF) mass spectrometer (all from Agilent Technologies, Santa Clara, CA, United States) via a JetStream dual electrospray ionization source. Chromatographic separation, source conditions and QTOF instrumental conditions were optimized following the methods in previous works^47,48^. Briefly, an Agilent Zorbax Eclipse Plus C18 column (100 × 2.1 mm, 1.8 μm) was used for separation. The mobile phase consisted of a binary mixture of methanol and water. The flow rate was set to 0.200 mL min^−1^ using a gradient of methanol (from 5 to 95%) within 34 min. The injection volume was 6 μL and acquisition was carried out in positive full-scan mode, detecting mass features in the range 100–1200 *m/z*. Samples were acquired in “extended dynamic range” mode with a nominal resolution of 40,000 FWHM.

The raw features from UHPLC-ESI/QTOF were processed using Profinder software (version B.07, from Agilent Technologies). The find-by-formula algorithm was used to annotate molecular features following mass and retention time alignment. The minimum absolute abundance was set to 8000 counts, the mass accuracy was 5 ppm and the isotope model of “common organic molecules” was chosen. The list of possible molecular formulae was provided by considering their accurate monoisotopic masses (error ≤ 5 ppm) and isotopic patterns (i.e., isotopic distribution, space and abundance). These latter were compared to those registered in the comprehensive database “MetaCyc”, which was used for annotation purposes. MetaCyc^11^ (www.metacyc.org) is a non-redundant reference database of small molecules containing experimentally verified metabolic pathways and enzyme information curated from the scientific literature^49^. Features were aligned (mass tolerance window: 5 ppm + 2mDa; retention time tolerance: 0.15 min) and retained if present in 100% of replicates in at least one condition. This post-acquisition filtering-by-frequency process allowed achieving higher confidence for the features that were actually present. Finally, the data were log2 transformed and centered to the median of the individual features in the dataset. This was done to treat all compounds equally regardless of their abundance.

### Chemometrics and statistics

Analysis of variance (one-way ANOVA) was carried out using PASW Statistics 25.0 (SPSS Inc.) to check for significant differences (p < 0.05) in the MDA content of *B. breve* DSM 20213 + LA vs *B. breve* DSM 20213. Afterward, the metabolomics data were elaborated in MPP following annotations. The abundance of metabolites was normalized at the 75^th^ percentile and then volcano plot analysis was carried out by combining fold-change (FC) analysis (cut-off = 2) and ANOVA (p < 0.01, Bonferroni multiple testing correction). Thereafter, an unsupervised statistical approach, Hierarchical Cluster Analysis (HCA), was used as previously described^47^. Furthermore, the raw metabolomics-based dataset was loaded into SIMCA 13 (Umetrics, Malmo, Sweden) for the supervised OPLS-DA approach. Confidence limits of 95% and 99% were used to check for the presence of outliers (suspect and strong outliers, respectively, according to Hotelling’s T2 approach), while cross-validation (CV-ANOVA, p < 0.01) and a permutation test (N = 500) to exclude overfitting were also carried out. The goodness-of-fit and prediction ability of the OPLS-DA model (i.e., R^2^Y and Q^2^Y, respectively) were also checked, adopting cut-off values for Q^2^Y = 0.5, as stated in the literature^50^. Finally, the VIP variable selection method (i.e., variable importance in projection) was carried out to identify those metabolites with the highest discrimination potential. Generally, according to software recommendations (Umetrics, Malmo, Sweden), VIP markers larger than 0.8 were considered the most meaningful in the prediction model. Finally, a smart table was created in MetaCyc (www.metacyc.org) using a targeted list of compounds obtained when considering those VIP markers possessing FC values > 2. The smart table was then loaded onto the “omics-dashboard”^15^ of the online tool in order to point out the pathways and processes most affected by the addition of LA to *B. breve* DSM 20213.

## Supplementary information

Supplementary Information.

Supplementary Information2.
