# Supplementary material for: Linoleic acid induces metabolic stress in the intestinal microorganism Bifidobacterium breve DSM 20213
Source: Sci Rep. 2020 Apr 7;10:5997. doi: 10.1038/s41598-020-62897-w (PMC7138814; doi:10.1038/s41598-020-62897-w)
Supplement: Supplementary file 1 — Supplementary Information. [file 41598_2020_62897_MOESM1_ESM.pdf]

Linoleic acid induces metabolic stress in the intestinal microorganism *Bifidobacterium breve* DSM 20213

Alice Senizza[1], Gabriele Rocchetti[1], Maria Luisa Callegari[1,2], Luigi Lucini[1], Lorenzo Morelli[1]

[1]*Department for Sustainable Food Process, Università Cattolica del Sacro Cuore, via Emilia Parmense 84, 29122 Piacenza, Italy*
[2]*Centre for Research on Biotechnology (CRB), Università Cattolica del Sacro Cuore, via Milano 24, 26100 Cremona, Italy*

SUPPLEMENTARY MATERIAL:

Supplementary File 2. Bar plot considering *B. breve* DSM 20213 + LA *vs. B. breve* DSM metabolic pathways from the Pathway Tools Omics Dashboard of MetaCyc (www.metacyc.org).

Supplementary File 3. VIP markers identified by OPLS-DA multivariate analysis of UHPLC-ESI-QTOF mass spectrometry data gained from *B. breve* DSM 20213 + LA and *B. breve* DSM 20213, as imported into MetaCyc omic viewer. LogFC values for each marker at different stages of the bacterial growth curve are also provided.

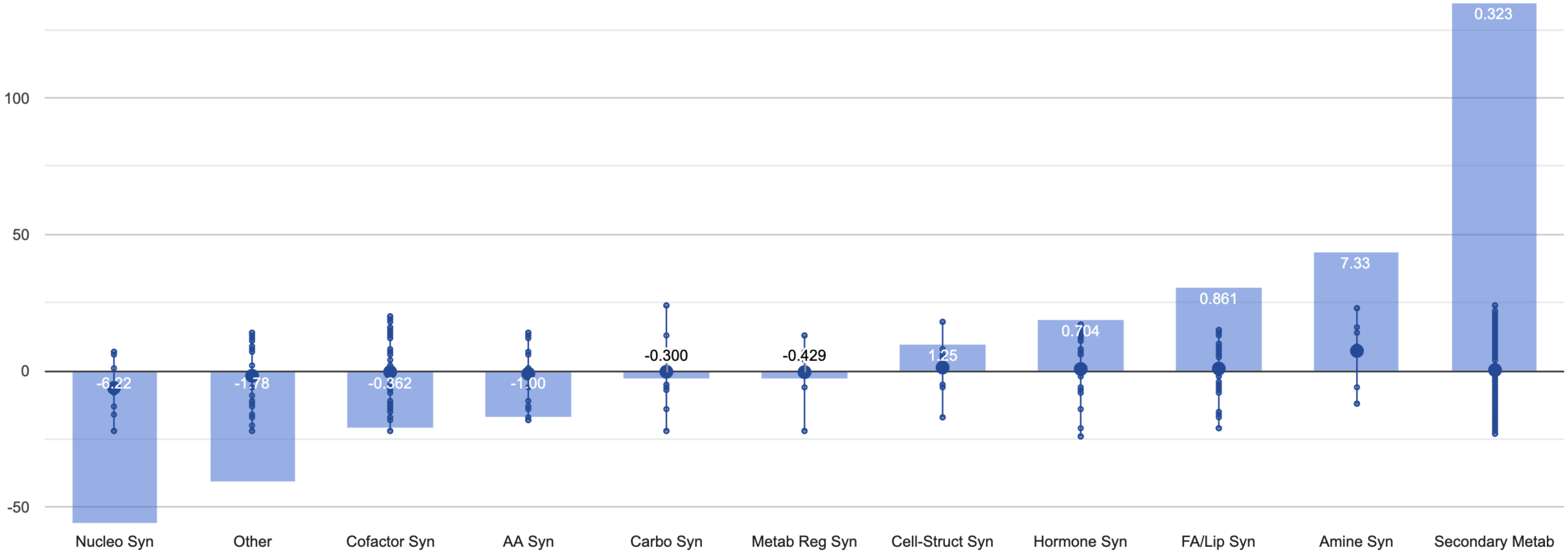

Karp P.D., Latendresse M., Paley S.M., Krummenacker M., Ong Q., Billington R., Kothari A., Weaver D., Lee T., Subhraveti P., Spaulding A., Fulcher C.A., Keseler I.M., Caspi R.
Pathway Tools version 19.0: Integrated Software for Pathway/Genome Informatics and Systems Biology
Briefings in Bioinformatics doi: 10.1093/bib/bbv079. (2015)

Metabolite changes, lag and log phases

Biochemical class	Metacyc code	Compound name	fold-change<br>LA-treated vs control, lag phase	LA-treated vs control, log phase
Amino Acid Biosynthesis	VAL	L-valine	0	-18
Amino Acid Biosynthesis	LEU	L-leucine	0	0
Amino Acid Biosynthesis	HIS	L-histidine	0	-18
Amino Acid Biosynthesis	PHE	L-phenylalanine	0	-16
Amino Acid Biosynthesis	L-HISTIDINOL-P	L-histidinol phosphate	2	2
Amino Acid Biosynthesis	NADH	NADH	17	0
Amino Acid Biosynthesis	ILE	L-isoleucine (ile)	0	-20
Amino Acid Biosynthesis	PAPS	3'-phosphoadenylyl-sulfate (PAPS)	15	-2
Nucleoside and Nucleotide Biosynthesis	GUANINE	guanine	-2	-20
Nucleoside and Nucleotide Biosynthesis	DATP	dATP	15	0
Nucleoside and Nucleotide Biosynthesis	DUTP	dUTP	17	0
Nucleoside and Nucleotide Biosynthesis	TTP	dTTP	0	-17
Nucleoside and Nucleotide Biosynthesis	DCTP	dCTP	15	-2
Nucleoside and Nucleotide Biosynthesis	NADH	NADH	17	0
Fatty Acid and Lipid Biosynthesis	CHOLINE	choline	0	-18
Fatty Acid and Lipid Biosynthesis	CPD-5441	N-dimethylethanolamine phosphate	0	8
Fatty Acid and Lipid Biosynthesis	LINOLEIC_ACID	linoleate	22	3
Fatty Acid and Lipid Biosynthesis	CPD-8477	petroselinate	18	2
Fatty Acid and Lipid Biosynthesis	OLEATE-CPD	oleate	20	2
Fatty Acid and Lipid Biosynthesis	STEARIC_ACID	stearate	-1	-19
Fatty Acid and Lipid Biosynthesis	18-OXOOLEATE	18-oxo-oleate	19	2
Fatty Acid and Lipid Biosynthesis	DEOXYCHOLATE	deoxycholate	-21	-36
Fatty Acid and Lipid Biosynthesis	CPD-481	sphingosylphosphorylcholine	-21	-18
Fatty Acid and Lipid Biosynthesis	GLYCOCHOLIC_ACID	glycocholate	-1	-18
Fatty Acid and Lipid Biosynthesis	CPD-7283	taurochenodeoxycholate	0	-18
Fatty Acid and Lipid Biosynthesis	CPD-3743	taurocholate	0	0
Fatty Acid and Lipid Biosynthesis	CPD-10538	digiproside	18	-37
Fatty Acid and Lipid Biosynthesis	CPD-18694	C36-phenolphthiodiolone A	17	-1
Fatty Acid and Lipid Biosynthesis	CPD-18658	C37-phenolphthiocerol A	-1	-29
Fatty Acid and Lipid Biosynthesis	CPD-465	presqualene diphosphate	16	-18
Fatty Acid and Lipid Biosynthesis	NADH	NADH	17	0
Fatty Acid and Lipid Biosynthesis	CPD-18625	(behenoyl)adenylate	0	-18
Fatty Acid and Lipid Biosynthesis	CPD1G-69	D-mannopyranosyl-1-phosphoheptaprenol	0	-17
Fatty Acid and Lipid Biosynthesis	CPD-8078	1-18:3-2-16:2-monogalactosyldiacylglycerol	0	-1
Fatty Acid and Lipid Biosynthesis	CPD-8086	1-linoleoyl-2-palmitoyl-phosphatidylglycerol	15	-1
Fatty Acid and Lipid Biosynthesis	CPD-2190	1-18:3-2-16:3-monogalactosyldiacylglycerol	15	0
Fatty Acid and Lipid Biosynthesis	CPD-2183	1-oleoyl-2-palmitoyl-phosphatidylglycerol	15	0
Fatty Acid and Lipid Biosynthesis	CPD-8079	1-18:1-2-16:3-monogalactosyldiacylglycerol	16	0
Fatty Acid and Lipid Biosynthesis	CPD-8076	1-18:3-2-16:1-monogalactosyldiacylglycerol	4	0
Fatty Acid and Lipid Biosynthesis	CPD-8167	1-18:3-2-18:2-monogalactosyldiacylglycerol	18	2
Fatty Acid and Lipid Biosynthesis	CPD-2188	1-18:1-2-16:1-monogalactosyldiacylglycerol	18	2
Fatty Acid and Lipid Biosynthesis	CPD-8165	1-18:2-2-18:2-monogalactosyldiacylglycerol	0	-19
Fatty Acid and Lipid Biosynthesis	CPD-2187	1-18:1-2-16:0-monogalactosyldiacylglycerol	0	-19
Fatty Acid and Lipid Biosynthesis	CPD-8158	1-palmitoyl-2-linoleoyl-phosphatidylcholine	9	0
Fatty Acid and Lipid Biosynthesis	CPD-12644	1-palmitoyl-2-vernoloyl-phosphatidylcholine	0	8
Fatty Acid and Lipid Biosynthesis	CPD-8162	1-18:3-2-16:0-digalactosyldiacylglycerol	17	0
Fatty Acid and Lipid Biosynthesis	CPD-8081	1-18:3-2-18:3-digalactosyldiacylglycerol	10	-1
Fatty Acid and Lipid Biosynthesis	CPD-8082	1-18:2-2-18:2-digalactosyldiacylglycerol	18	0
Fatty Acid and Lipid Biosynthesis	Vernolates	vernolate	19	2
Fatty Acid and Lipid Biosynthesis	PAPS	3'-phosphoadenylyl-sulfate (PAPS)	15	-2
Amine and Polyamine Biosynthesis	CHOLINE	choline	0	-18
Amine and Polyamine Biosynthesis	BETAINE	glycine betaine	0	-18
Amine and Polyamine Biosynthesis	TYRAMINE	tyramine	0	-17
Amine and Polyamine Biosynthesis	CREATINE	creatine	16	-11
Amine and Polyamine Biosynthesis	HIS	L-histidine	0	-18
Amine and Polyamine Biosynthesis	CPD-5441	N-dimethylethanolamine phosphate	0	8
Amine and Polyamine Biosynthesis	NADH	NADH	17	0
Secondary Metabolite Biosynthesis	CPD-11528	3-oxo-2-(cis-2'-pentenyl)-cyclopentane-1-(3-oxo	17	0
Secondary Metabolite Biosynthesis	CPD-2482	D-cycloserine	0	-17
Secondary Metabolite Biosynthesis	CPD-6361	indolin-2-one	-18	-18
Secondary Metabolite Biosynthesis	CPD-8853	2-descarboxy-cyclo-dopa	0	-18
Secondary Metabolite Biosynthesis	BACIMETHRIN	bacimethrin	0	-18
Secondary Metabolite Biosynthesis	CPD-10211	furaneol (enol form)	0	8
Secondary Metabolite Biosynthesis	CPD-2205	(E)-(4-hydroxyphenyl)acetaldehyde oxime	-18	-18
Secondary Metabolite Biosynthesis	CPD-8914	(1H-indol-3-yl)-N-methylmethanamine	0	-19
Secondary Metabolite Biosynthesis	1-7-DIMETHYLXANTHINE	paraxanthine	2	2
Secondary Metabolite Biosynthesis	CPD-18776	(R)-4-deoxygadusol	0	-16
Secondary Metabolite Biosynthesis	D-GLUCARATE	D-glucarate	-17	-18
Secondary Metabolite Biosynthesis	CPD-6963	dihydropinosylvin	0	-18
Secondary Metabolite Biosynthesis	CPD-18032	fusarinolate	0	-7
Secondary Metabolite Biosynthesis	CPD-9940	harmine	0	-7
Secondary Metabolite Biosynthesis	INDOLE_PYRUVATE	(indol-3-yl)pyruvate	2	2
Secondary Metabolite Biosynthesis	6-AMINOPENICILLANATE	6-aminopenicillanate	0	-7
Secondary Metabolite Biosynthesis	CPD-398	(R)-3-(indol-3-yl)-2-oxobutanoate	0	-7
Secondary Metabolite Biosynthesis	CPD-17043	cyclo(L-phenylalanyl-L-seryl)	-22	-18
Secondary Metabolite Biosynthesis	CPD-13616	pentalen-13-ol	0	-18
Secondary Metabolite Biosynthesis	CPD-4744	3-hydroxylubimin	-17	-19
Secondary Metabolite Biosynthesis	CPD-14471	elymoclavine aldehyde	0	0
Secondary Metabolite Biosynthesis	CPD-4742	2-dehydrolubimin	-17	-19
Secondary Metabolite Biosynthesis	CPD-19419	norcraugsodine	0	-1
Secondary Metabolite Biosynthesis	CPD-9366	(-)-vestitol	18	-18
Secondary Metabolite Biosynthesis	CPD-13619	pentalenolactone	-22	-18
Secondary Metabolite Biosynthesis	CPD-12973	demethyldehydrophos	-21	0

Secondary Metabolite Biosynthesis	CPD-12674	salinosporamide A	-21	0
Secondary Metabolite Biosynthesis	CPD-17539	dapdiamide A	-1	-19
Secondary Metabolite Biosynthesis	CPD-19429	hemanthamine	-1	-19
Secondary Metabolite Biosynthesis	CPD-13541	kauralexin A3	19	3
Secondary Metabolite Biosynthesis	CPD-14815	protoemetine	17	-9
Secondary Metabolite Biosynthesis	CPD-1777	coniferin	-1	-19
Secondary Metabolite Biosynthesis	CPD-18058	prefusarin	-19	0
Secondary Metabolite Biosynthesis	CPD-17992	equisetin	-19	0
Secondary Metabolite Biosynthesis	CPD-12949	UWM6	0	0
Secondary Metabolite Biosynthesis	CPD-15906	aurachin RE	-4	-20
Secondary Metabolite Biosynthesis	CPD-14629	spinosyn tricyclic macrolactone	-17	-18
Secondary Metabolite Biosynthesis	CPD-17334	preandiloid B	-1	-18
Secondary Metabolite Biosynthesis	CPD-12600	2-arachidonoylglycerol	-1	0
Secondary Metabolite Biosynthesis	CPD-19790	adlupulone	-11	-27
Secondary Metabolite Biosynthesis	CPD-7110	lupulone	-21	-38
Secondary Metabolite Biosynthesis	CPD-14627	15-oxo spinosyn macrolactone	-1	-18
Secondary Metabolite Biosynthesis	CPD-16743	13-desoxypaxilline	-1	-18
Secondary Metabolite Biosynthesis	CPD-17361	notoamide D	17	0
Secondary Metabolite Biosynthesis	CPD-7973	bixin dimethyl ester	0	-17
Secondary Metabolite Biosynthesis	CPD-18646	furostanol-26-alcohol	-24	-18
Secondary Metabolite Biosynthesis	CPD-16956	terpendole E	-1	-18
Secondary Metabolite Biosynthesis	CPD-14818	7'-O-demethylcephaeline	0	-1
Secondary Metabolite Biosynthesis	CPD-17192	terpendole F	16	-1
Secondary Metabolite Biosynthesis	CPD-18332	lyngbyatoxin C	-22	0
Secondary Metabolite Biosynthesis	CPD-13830	10-deoxymethymycin	-23	-18
Secondary Metabolite Biosynthesis	CPD-14502	steviolmonoside	10	-2
Secondary Metabolite Biosynthesis	CPD-9470	gypsogenin	-21	0
Secondary Metabolite Biosynthesis	CPD-14206	gentamicin A	7	-9
Secondary Metabolite Biosynthesis	TTP	dTTP	0	-17
Secondary Metabolite Biosynthesis	CPD-18226	guadinomine B	0	-18
Secondary Metabolite Biosynthesis	CPD-14215	JI-20A	-1	-18
Secondary Metabolite Biosynthesis	CPD-15998	novamethymycin	-1	-18
Secondary Metabolite Biosynthesis	CPD-16839	fusicoccin P	0	-18
Secondary Metabolite Biosynthesis	CPD-17431	prochaetoglobosin IV	0	-18
Secondary Metabolite Biosynthesis	CPD-10076	3'-keto-3'-dehydroATP	0	-17
Secondary Metabolite Biosynthesis	CPD-16963	aflatrem	-1	-18
Secondary Metabolite Biosynthesis	CPD-9483	medicagenate	-3	-19
Secondary Metabolite Biosynthesis	CPD-9474	gypsogenate	5	-17
Secondary Metabolite Biosynthesis	CPD-14817	emetine	-1	-18
Secondary Metabolite Biosynthesis	CPD-13835	pikromycin	-1	-18
Secondary Metabolite Biosynthesis	CPD-14214	gentamicin X2	-1	-18
Secondary Metabolite Biosynthesis	CPD-17429	cytoglobosin D	0	-18
Secondary Metabolite Biosynthesis	CPD-13834	narbomycin	18	0
Secondary Metabolite Biosynthesis	CPD-17197	terpendole J	0	-17
Secondary Metabolite Biosynthesis	CPD-14630	rhamnosyl tricyclic spinosyn pseudoaglycone	8	0
Secondary Metabolite Biosynthesis	CPD-14631	rhamnosyl tetracyclic spinosyn pseudoaglycone	-2	-19
Secondary Metabolite Biosynthesis	CPD-13825	L-oleandrosyl-oleandolide	-1	-18
Secondary Metabolite Biosynthesis	CPD-9635	hyperforin	0	-18
Secondary Metabolite Biosynthesis	CPD-12232	hordatine A	-1	-18
Secondary Metabolite Biosynthesis	UDP-APIOSE	UDP-D-apiose	0	-17
Secondary Metabolite Biosynthesis	CPD-14520	2-methylbacteriohopanetetrol	17	-1
Secondary Metabolite Biosynthesis	CPD-21021	acinetoferrin	0	-18
Secondary Metabolite Biosynthesis	CPD-15996	novapikromycin	0	-18
Secondary Metabolite Biosynthesis	CPD-3764	desferrioxamine B	0	-18
Secondary Metabolite Biosynthesis	CPD-15033	nostoxanthin	-1	-18
Secondary Metabolite Biosynthesis	CPD-20680	diadinoxanthin	0	-18
Secondary Metabolite Biosynthesis	CPD-15032	caloxanthin	0	-18
Secondary Metabolite Biosynthesis	CPD-17660	2-hydroxycanthaxanthin	16	0
Secondary Metabolite Biosynthesis	CPD-465	presqualene diphosphate	16	-18
Secondary Metabolite Biosynthesis	CPD-17731	linoleyl-AMP	-1	0
Secondary Metabolite Biosynthesis	CPD-17665	2-hydroxyastaxanthin	0	-1
Secondary Metabolite Biosynthesis	CPD-20691	amarouciaxanthin A	0	0
Secondary Metabolite Biosynthesis	CPD-20690	fucoxanthinol	17	0
Secondary Metabolite Biosynthesis	CPD-14142	neomycin B	0	-1
Secondary Metabolite Biosynthesis	CPD-17204	lolitriol	0	-1
Secondary Metabolite Biosynthesis	CPD-14177	paromomycin	0	-1
Secondary Metabolite Biosynthesis	CPD-17957	apicidin	0	-18
Secondary Metabolite Biosynthesis	CPD-9472	gypsogenin-28-beta-D-glucoside	17	0
Secondary Metabolite Biosynthesis	CPD-9475	gypsogenate-28-beta-D-glucoside	0	-18
Secondary Metabolite Biosynthesis	CPD-20679	fucoxanthin	0	0
Secondary Metabolite Biosynthesis	CPD-14522	adenosyl hopane	18	1
Secondary Metabolite Biosynthesis	CPD-9477	16-alpha-hydroxygypsogenate-28-beta-D-glucos	0	-1
Secondary Metabolite Biosynthesis	CPD-13823	mycinamicin VI	0	-18
Secondary Metabolite Biosynthesis	CPD-12803	fuscicoccin A	0	-1
Secondary Metabolite Biosynthesis	CPD-17202	lolitrem B	0	0
Secondary Metabolite Biosynthesis	CPD-8658	amaranthin	0	-1
Secondary Metabolite Biosynthesis	CPD-17500	decarboxy-dehydroxy-demycosaminyl-nystatin	0	-17
Secondary Metabolite Biosynthesis	CPD-13805	erythromycin B	0	-1
Secondary Metabolite Biosynthesis	CPD-464	prephytoene diphosphate	0	-17
Secondary Metabolite Biosynthesis	CPD-15297	lampranthin II	0	-1
Secondary Metabolite Biosynthesis	CPD-17498	demycosaminyl-nystatin	-3	-19
Secondary Metabolite Biosynthesis	CPD-9916	staphyloxanthin	0	-18
Secondary Metabolite Biosynthesis	CPD-16625	monodeglucosyl des-acyl avenacin A	0	-17
Secondary Metabolite Biosynthesis	CPD-12680	4-chloro-crotonyl-CoA	19	2
Secondary Metabolite Biosynthesis	TRANS-3-METHYL-GLUTACONYL	3-methylglutaconyl-CoA	18	-1
Secondary Metabolite Biosynthesis	CPD-12675	chloroethylmalonyl-CoA	18	1
Secondary Metabolite Biosynthesis	CPD-514	3-oxo-3-phenylpropanoyl-CoA	16	0
Secondary Metabolite Biosynthesis	CPD-9772	4-(1-methyl-2-pyrrolidinyl)-3-oxobutanoyl-CoA	0	0
Secondary Metabolite Biosynthesis	CPD-12180	feruloylacetyl-CoA	8	0
Secondary Metabolite Biosynthesis	P-COUMAROYL-COA	(E)-4-coumaroyl-CoA	16	0
Secondary Metabolite Biosynthesis	CPDQT-35	2-[(7'-methylsulfanyl)heptyl]malate	-19	-18

Secondary Metabolite Biosynthesis	PAPS	3'-phosphoadenylyl-sulfate (PAPS)	15	-2
Secondary Metabolite Biosynthesis	CPD-4663	3-deoxycapsidiol	0	-18
Secondary Metabolite Biosynthesis	MI-HEXAKISPHOSPHATE	phytate (Ins(1,2,3,4,5,6)	18	0
Secondary Metabolite Biosynthesis	CPD-8728	isopimara-7,15-dienal	-1	-28
Secondary Metabolite Biosynthesis	AMINO-RIBOSYLAMINO-1H-3H-F	5-amino-6-(D-ribitylamino)uracil (ARP)	-19	-18
Cofactor	CPD-15779	2-(2-methylpyridin-3-yl)ethanol	0	-17
Cofactor	TYRAMINE	tyramine	0	-17
Cofactor	2-DEHYDROPANTOYL-LACTONE	2-dehydropantolactone	0	8
Cofactor	CPD-13577	formylaminopyrimidine	0	-17
Cofactor	CPD-7323	methanophenazine	-1	-18
Cofactor	CPD-8124	thio-molybdenum cofactor	0	-17
Cofactor	2-HEXAPRENYL-3-METHYL-5-HYD	3-demethylubiquinol-6	0	-18
Cofactor	UBIQUINOL-30	ubiquinol-6	0	0
Cofactor	CPD-9852	3-heptaprenyl-4-hydroxybenzoate	0	-1
Cofactor	CPDQT-400	ADP-5-ethyl-4-methylthiazole-2-carboxylate	0	0
Cofactor	CPD-12117	demethylmenaquinol-7	16	0
Cofactor	NADH	NADH	17	0
Cofactor	DEAMIDO-NAD	nicotinate adenine dinucleotide	17	0
Cofactor	CPD-17070	Fe-coproporphyrin III	0	0
Cofactor	3-OCTAPRENYL-4-HYDROXYBENZ	3-octaprenyl-4-hydroxybenzoate	17	1
Cofactor	NONAPRENYL-4-HYDROXYBENZO	3-nonaprenyl-4-hydroxybenzoate	0	-1
Cofactor	CPD-12118	demethylmenaquinol-9	17	0
Cofactor	AMINO-RIBOSYLAMINO-1H-3H-F	5-amino-6-(D-ribitylamino)uracil (ARP)	-19	-18
Cofactor	PAPS	3'-phosphoadenylyl-sulfate (PAPS)	15	-2
Cofactor	CPD-15190	3-all trans-hexaprenyl-4-aminobenzoate	15	-7
Cofactor	CPD-15191	3-all trans-hexaprenyl-4-amino-5-hydroxybenzo	0	-18
Cell Structure Biosynthesis	CHOLINE	choline	0	-18
Cell Structure Biosynthesis	TYRAMINE	tyramine	0	-17
Cell Structure Biosynthesis	PHE	L-phenylalanine	0	-16
Cell Structure Biosynthesis	18-HYDROXYOLEATE	18-hydroxyoleate	22	3
Cell Structure Biosynthesis	OLEATE-CPD	oleate	20	2
Cell Structure Biosynthesis	CPD-17640	18-hydroxystearate	19	1
Cell Structure Biosynthesis	STEARIC_ACID	stearate	-1	-19
Cell Structure Biosynthesis	18-OXOOLEATE	18-oxo-oleate	19	2
Cell Structure Biosynthesis	NADH	NADH	17	0
Cell Structure Biosynthesis	P-COUMAROYL-COA	(E)-4-coumaroyl-CoA	16	0
Metabolic Regulator Biosynthesis	HIS	L-histidine	0	-18
Metabolic Regulator Biosynthesis	CREATINE	creatine	16	-11
Metabolic Regulator Biosynthesis	NADH	NADH	17	0
Metabolic Regulator Biosynthesis	CARNITINE	L-carnitine	0	-18
Metabolic Regulator Biosynthesis	GDP-TP	pppGpp	17	-1

Metabolite changes, stationary phase

Biochemical class	Metacyc code	Compound name	fold-change LA-treated vs control, stationary phase
Amino Acid Biosynthesis	CYS	L-cysteine	13
Amino Acid Biosynthesis	ACETYL-COA	acetyl-CoA	-6
Amino Acid Biosynthesis	2-AMINOACRYLATE	2-aminoprop-2-enoate	-17
Amino Acid Biosynthesis	O-SUCCINYL-L-HOMOSERINE	O-succinyl-L-homoserine	6
Amino Acid Biosynthesis	MET	L-methionine	7
Amino Acid Biosynthesis	HISTIDINOL	histidinol	-1
Amino Acid Biosynthesis	IMIDAZOLE-ACETOL-P	3-(imidazol-4-yl)-2-oxopropyl phosphate	-2
Amino Acid Biosynthesis	D-ERYTHRO-IMIDAZOLE-GLYCEROL-P	D-erythro-1-(imidazol-4-yl)-glycerol 3-phosphate	-2
Amino Acid Biosynthesis	IMIDAZOLE-LACTATE	imidazole-lactate	-14
Amino Acid Biosynthesis	CPD-31	(R)-citramalate	-13
Amino Acid Biosynthesis	SUC-COA	succinyl-CoA	7
Amino Acid Biosynthesis	CPD-68	1-aminocyclopropane-1-carboxylate	7
Amino Acid Biosynthesis	SHIKIMATE-5P	shikimate 3-phosphate	-11
Amino Acid Biosynthesis	TYR	L-tyrosine	14
Amino Acid Biosynthesis	CARBOXYPHENYLAMINO-DEOXYRIBULOSE-P	1-(2-carboxyphenylamino)-1-deoxy-D-ribulose 5-phospha	-18
Amino Acid Biosynthesis	TRP	L-tryptophan (trp)	12
Amino Acid Biosynthesis	INDOLE	indole	1
Nucleoside and Nucleotide Biosynthesis	XANTHINE	xanthine	-8
Nucleoside and Nucleotide Biosynthesis	XANTHOSINE	xanthosine	7
Nucleoside and Nucleotide Biosynthesis	INOSINE	inosine	-13
Nucleoside and Nucleotide Biosynthesis	PHOSPHORIBOSYL-FORMAMIDO-CARBOXAMIDE	5-formamido-1-(5-phospho-D-ribosyl)-imidazole-4-carbo	-6
Nucleoside and Nucleotide Biosynthesis	GTP	GTP	-22
Nucleoside and Nucleotide Biosynthesis	TTP	dTTP	1
Nucleoside and Nucleotide Biosynthesis	TDP	dTDP	-5
Nucleoside and Nucleotide Biosynthesis	UMP	UMP	6
Nucleoside and Nucleotide Biosynthesis	OROTATE	orotate	-16
Fatty Acid and Lipid Biosynthesis	ACETYL-COA	acetyl-CoA	-6
Fatty Acid and Lipid Biosynthesis	CPD-10512	16-oxo-palmitate	-6
Fatty Acid and Lipid Biosynthesis	TDP	dTDP	-5
Fatty Acid and Lipid Biosynthesis	CPD-12644	1-palmitoyl-2-vernoloyl-phosphatidylcholine	2
Fatty Acid and Lipid Biosynthesis	DODECANOATE	laurate	8
Fatty Acid and Lipid Biosynthesis	CPD-7836	myristate	13
Fatty Acid and Lipid Biosynthesis	ARACHIDONIC_ACID	arachidonate	-2
Fatty Acid and Lipid Biosynthesis	CPD-9245	palmitoleate	-16
Fatty Acid and Lipid Biosynthesis	CPD-8073	1-18:2-2-16:0-monogalactosyldiacylglycerol	9
Fatty Acid and Lipid Biosynthesis	CPD-8075	1-18:2-2-16:1-monogalactosyldiacylglycerol	-7
Fatty Acid and Lipid Biosynthesis	CPD-8163	1-16:0-2-18:2-digalactosyldiacylglycerol	15
Fatty Acid and Lipid Biosynthesis	CPD-8168	1-18:3-2-18:3-monogalactosyldiacylglycerol	14
Fatty Acid and Lipid Biosynthesis	UMP	UMP	6
Fatty Acid and Lipid Biosynthesis	GLYCEROL	glycerol	5
Fatty Acid and Lipid Biosynthesis	CPD0-1812	2-oleoylglycerol	9
Fatty Acid and Lipid Biosynthesis	CPD-18709	(palmitoyl)adenylate	-8
Fatty Acid and Lipid Biosynthesis	CPD-18617	p-HBAD-I	-4
Fatty Acid and Lipid Biosynthesis	CPD-18623	17-(4-hydroxyphenyl)heptadecanoate	14
Fatty Acid and Lipid Biosynthesis	CPD-18655	C34-phenolphthiodiolone A	9
Fatty Acid and Lipid Biosynthesis	CPD-18654	C34-phenolphthiotriol A	-8
Fatty Acid and Lipid Biosynthesis	CPD-18670	C35-phenolcarboxyphthiodiolenone	7
Fatty Acid and Lipid Biosynthesis	CPD-18671	C37-phenolcarboxyphthiodiolenone	1
Fatty Acid and Lipid Biosynthesis	CPD-18660	C36-phenolphthiodiolenone A	10
Fatty Acid and Lipid Biosynthesis	CPD-18659	19-(4-hydroxyphenyl)nonadecanoyl adenylate	-7
Fatty Acid and Lipid Biosynthesis	CPD-8086	1-linoleoyl-2-palmitoyl-phosphatidylglycerol	15
Fatty Acid and Lipid Biosynthesis	CPD-8091	1-oleoyl-2-linoleoyl-phosphatidylcholine	1
Fatty Acid and Lipid Biosynthesis	CPD-2182	1-linoleoyl-2-linoleoyl-phosphatidylcholine	-15
Fatty Acid and Lipid Biosynthesis	CPD-18629	C33-phthiodiolone A	-6
Fatty Acid and Lipid Biosynthesis	CPD-18630	C33-phthiotriol A	13
Fatty Acid and Lipid Biosynthesis	CPD-13611	icosasphinganine	7
Fatty Acid and Lipid Biosynthesis	SPHINGOSINE	sphingosine	15
Fatty Acid and Lipid Biosynthesis	CPD-14901	poriferst-7-enol	-16
Fatty Acid and Lipid Biosynthesis	CPD-3743	taurocholate	8
Fatty Acid and Lipid Biosynthesis	CPD-7283	taurochenodeoxycholate	-17
Fatty Acid and Lipid Biosynthesis	CPD-707	campesterol	-21
Fatty Acid and Lipid Biosynthesis	CPD-4181	cycloartanol	-1
Fatty Acid and Lipid Biosynthesis	3-OXO-5-BETA-CHOLANATE	3-dehydrolithocholate	8
Amine and Polyamine Biosynthesis	BETAINE-ALDEHYDE-HYDRATE	betaine aldehyde hydrate	-12
Amine and Polyamine Biosynthesis	ACETYL-COA	acetyl-CoA	-6
Amine and Polyamine Biosynthesis	CPD-9995	carboxynorspermidine	16
Amine and Polyamine Biosynthesis	TYR	L-tyrosine	14
Amine and Polyamine Biosynthesis	CPD-58	octopamine	9
Amine and Polyamine Biosynthesis	CPD-10012	carboxyspermidine	23
Carbohydrate Biosynthesis	L-SORBOSONE	L-sorbosone	-14
Carbohydrate Biosynthesis	UMP	UMP	6
Carbohydrate Biosynthesis	TDP	dTDP	-5
Carbohydrate Biosynthesis	ACETYL-COA	acetyl-CoA	-6
Carbohydrate Biosynthesis	GTP	GTP	-22
Carbohydrate Biosynthesis	DPG	3-phospho-D-glyceroyl phosphate (13-DPG)	13
Carbohydrate Biosynthesis	CPD-464	prephytoene diphosphate	-7
Carbohydrate Biosynthesis	MET	L-methionine	7
Carbohydrate Biosynthesis	TTP	dTTP	1
Carbohydrate Biosynthesis	CPD-17206	GDP-mycosamine	24
Secondary Metabolite Biosynthesis	CPD-17074	(-)-microperfuranone	16
Secondary Metabolite Biosynthesis	TRP	L-tryptophan (trp)	12
Secondary Metabolite Biosynthesis	SUC-COA	succinyl-CoA	7
Secondary Metabolite Biosynthesis	CPD-7901	2-tridecanone	7
Secondary Metabolite Biosynthesis	CPD-10227	1-deoxy-1-imino-D-erythrose 4-phosphate	4
Secondary Metabolite Biosynthesis	STERIGMATOCYSTIN	sterigmatocystin	-20
Secondary Metabolite Biosynthesis	CPD-16743	13-desoxypaxilline	15
Secondary Metabolite Biosynthesis	CPD-16963	aflatrem	-9
Secondary Metabolite Biosynthesis	CPD-17337	andilesin D	-14
Secondary Metabolite Biosynthesis	ACETYL-COA	acetyl-CoA	-6
Secondary Metabolite Biosynthesis	CPD-16792	rhizocticin B	6
Secondary Metabolite Biosynthesis	OLEANDOMYCIN	oleandomycin	15

Secondary Metabolite Biosynthesis	CPD-13826	L-olivosyl-oleandolide	8
Secondary Metabolite Biosynthesis	CPD-13827	oleandolide	22
Secondary Metabolite Biosynthesis	DEMETHYLMACROCIN	demethylmacrocin	7
Secondary Metabolite Biosynthesis	TYLOSIN	tylosin	20
Secondary Metabolite Biosynthesis	CPD-16508	L-(4-hydroxyphenyl)glycine	-18
Secondary Metabolite Biosynthesis	CPD-9418	nocardicin G	-6
Secondary Metabolite Biosynthesis	CPD-14209	G-418	-14
Secondary Metabolite Biosynthesis	CPD-14213	gentamicin C1	15
Secondary Metabolite Biosynthesis	CPD-14212	gentamicin C2a	8
Secondary Metabolite Biosynthesis	CPD-15229	3'-phospho-JI-20A	-7
Secondary Metabolite Biosynthesis	CPD-17208	18-hydroxy candicinolide	6
Secondary Metabolite Biosynthesis	CPD-17193	candicinolide	5
Secondary Metabolite Biosynthesis	CPD-19834	8-demethyl-8-(methylamino)riboflavin	-7
Secondary Metabolite Biosynthesis	CPD-12847	pyocyanin	7
Secondary Metabolite Biosynthesis	CPD-17531	L-dihydroanticapsin	9
Secondary Metabolite Biosynthesis	CPD-17508	bacilysin	1
Secondary Metabolite Biosynthesis	CPD-10013	bialaphos	-7
Secondary Metabolite Biosynthesis	CPD-18284	sch210971	8
Secondary Metabolite Biosynthesis	CPD-13831	10-deoxymethynolide	-7
Secondary Metabolite Biosynthesis	CPD-13830	10-deoxymethymycin	1
Secondary Metabolite Biosynthesis	CPD-13619	pentalenolactone	7
Secondary Metabolite Biosynthesis	CPD-13622	pentalenolactone F	-22
Secondary Metabolite Biosynthesis	CPD-20141	6'-chloromelleolide F	15
Secondary Metabolite Biosynthesis	CPD-12952	dehydrorabelomycin	-5
Secondary Metabolite Biosynthesis	CPD-14187	6'''-hydroxyparomomycin	1
Secondary Metabolite Biosynthesis	CPD-14188	6'''-oxoparomomycin	-14
Secondary Metabolite Biosynthesis	CPD-15934	protomycinolide IV	-14
Secondary Metabolite Biosynthesis	CPD-13820	mycinamicin III	-8
Secondary Metabolite Biosynthesis	CPD-13822	mycinamicin II	-7
Secondary Metabolite Biosynthesis	CPD-13821	mycinamicin IV	8
Secondary Metabolite Biosynthesis	CPD-17541	dapdiamide C	9
Secondary Metabolite Biosynthesis	CPD-17540	dapdiamide B	16
Secondary Metabolite Biosynthesis	CPD-15965	2-oxo-4-phosphonobutanoate	-16
Secondary Metabolite Biosynthesis	CPD-17786	griseofulvin	18
Secondary Metabolite Biosynthesis	CPD-11755	dichloro-arcyriaflavin A	-16
Secondary Metabolite Biosynthesis	CPD-11754	dichlorochromopyrrolate	-6
Secondary Metabolite Biosynthesis	CPD-17957	apicidin	4
Secondary Metabolite Biosynthesis	CPD-15531	5-hydroxymethylcytosine	1
Secondary Metabolite Biosynthesis	CPD0-2021	bacimethrin pyrophosphate	13
Secondary Metabolite Biosynthesis	CPDMETA-13642	neopentalenoketolactone	-10
Secondary Metabolite Biosynthesis	CPD-16513	saframycin A	1
Secondary Metabolite Biosynthesis	CPD-16512	3-hydroxy-5-methyl-L-tyrosine	-13
Secondary Metabolite Biosynthesis	CPD-18559	3-hydroxyquinaldate adenylate	6
Secondary Metabolite Biosynthesis	STREPTOMYCIN	streptomycin	2
Secondary Metabolite Biosynthesis	CPD-12972	[1-(2-amino-4-methylpentanamido)ethenyl]phosphonate	-4
Secondary Metabolite Biosynthesis	CPD-17787	griseophenone C	-20
Secondary Metabolite Biosynthesis	CPD-17799	desmethyl-dehydro-dechlorogriseofulvin	-14
Secondary Metabolite Biosynthesis	CPD-17800	dechloro-dehydrogriseofulvin	-7
Secondary Metabolite Biosynthesis	CPD-11887	K252a	4
Secondary Metabolite Biosynthesis	CPD-11889	K252b	13
Secondary Metabolite Biosynthesis	CPD-17158	arginomycin	6
Secondary Metabolite Biosynthesis	CPD-17164	L-leucyl-arginomycin	1
Secondary Metabolite Biosynthesis	CPD-13894	4-propyl-L-proline	20
Secondary Metabolite Biosynthesis	CPD-13835	pikromycin	-2
Secondary Metabolite Biosynthesis	CPD-18203	4-amino-2-(methanethioyl)-5-oxo-3-sulfanylpyrrolidine-2	-11
Secondary Metabolite Biosynthesis	CPD-17948	holothin	13
Secondary Metabolite Biosynthesis	CPD-15467	3-dimethylallyl-4-hydroxyphenylpyruvate	-12
Secondary Metabolite Biosynthesis	TYR	L-tyrosine	14
Secondary Metabolite Biosynthesis	CPD-18932	2-(phosphinatomethylidene)butanedioate	-16
Secondary Metabolite Biosynthesis	CPD-10011	demethyl-L-phosphinothricin	1
Secondary Metabolite Biosynthesis	CPD-18977	pseudomonate C	8
Secondary Metabolite Biosynthesis	CPD-7733	aurachin C	15
Secondary Metabolite Biosynthesis	CPD-15912	aurachin B epoxide	-15
Secondary Metabolite Biosynthesis	CPD-15909	aurachin D	17
Secondary Metabolite Biosynthesis	CPD-16638	12-demethyl-elloramycin	-6
Secondary Metabolite Biosynthesis	CPD-13100	toyocamycin phosphate	-17
Secondary Metabolite Biosynthesis	ANHYDROTETRACYCLINE	anhydrotetracycline	-6
Secondary Metabolite Biosynthesis	CPD0-1414	tetracycline	5
Secondary Metabolite Biosynthesis	CPD-17874	5-hydroxy-desmethylanthrotainin	11
Secondary Metabolite Biosynthesis	CPD-17871	previridicatumtoxin	-1
Secondary Metabolite Biosynthesis	CPD-11909	dTDP-L-ristosamine	16
Secondary Metabolite Biosynthesis	CPD-11789	K-252c	-14
Secondary Metabolite Biosynthesis	CPD-11786	chromopyrrolate	1
Secondary Metabolite Biosynthesis	CYS	L-cysteine	13
Secondary Metabolite Biosynthesis	CPD-18437	3-hydroxy-4-methyl-anthranilate pentapeptide lactone	9
Secondary Metabolite Biosynthesis	CPD-18448	3-hydroxy-4-methylanthranilyl-adenylate	-11
Secondary Metabolite Biosynthesis	CPD-17739	echinocandin D	18
Secondary Metabolite Biosynthesis	CPD-17711	echinocandin B	12
Secondary Metabolite Biosynthesis	CPD-17212	nystatin A1	13
Secondary Metabolite Biosynthesis	CPD-17206	GDP-mycosamine	24
Secondary Metabolite Biosynthesis	CPD-12966	megalomicin A	7
Secondary Metabolite Biosynthesis	CPD-13951	erythromycin C	8
Secondary Metabolite Biosynthesis	TTP	dTTP	1
Secondary Metabolite Biosynthesis	CPD-14156	2'-deamino-2'-hydroxy-6'-dehydroparomamine	-14
Secondary Metabolite Biosynthesis	CPD-14127	2'-deamino-2'-hydroxyparomamine	-7
Secondary Metabolite Biosynthesis	CPD-15254	2'-dehydrokanamycin A	-15
Secondary Metabolite Biosynthesis	CPD-4822	kanamycin B	15
Secondary Metabolite Biosynthesis	GTP	GTP	-22
Secondary Metabolite Biosynthesis	CPD-18787	GDP-valienol	7
Secondary Metabolite Biosynthesis	CPD-18790	validamycin B	8
Secondary Metabolite Biosynthesis	CPD-18226	guadinomine B	8
Secondary Metabolite Biosynthesis	CPD-15729	methyl aklanonate	5
Secondary Metabolite Biosynthesis	CPD-15733	13-deoxydaunorubicin	-13
Secondary Metabolite Biosynthesis	CPD-15735	13-dihydrodaunorubicin	4
Secondary Metabolite Biosynthesis	CPD-15736	13-dihydrocarminomycin	6
Secondary Metabolite Biosynthesis	AMIDINOPROCLAVAMINATE	amidinoproclavaminate	-15
Secondary Metabolite Biosynthesis	CPD-17169	L-leucyl-demethyl-blasticidin S	2
Secondary Metabolite Biosynthesis	CPD-17168	L-leucyl-blasticidin S	8
Secondary Metabolite Biosynthesis	CPD-10236	rifamycin SV	9

Secondary Metabolite Biosynthesis	CPD-16644	tetracenomycin B3	1
Secondary Metabolite Biosynthesis	UMP	UMP	6
Secondary Metabolite Biosynthesis	CPD-398	(R)-3-(indol-3-yl)-2-oxobutanoate	6
Secondary Metabolite Biosynthesis	CPD-18934	indolmycenate	16
Secondary Metabolite Biosynthesis	CPD0-920	indolmycin	8
Secondary Metabolite Biosynthesis	CPD-14167	6'''-deamino-6'''-oxoneomycin C	8
Secondary Metabolite Biosynthesis	CPD-10151	2-deoxystreptamine	7
Secondary Metabolite Biosynthesis	TDP	dTDP	-5
Secondary Metabolite Biosynthesis	CPD-428	6-deoxyerythronolide B	13
Secondary Metabolite Biosynthesis	CPD-9122	penicillin K	7
Secondary Metabolite Biosynthesis	DPG	3-phospho-D-glyceroyl phosphate (13-DPG)	13
Secondary Metabolite Biosynthesis	CPD-18237	zwittermicin A	8
Secondary Metabolite Biosynthesis	MET	L-methionine	7
Secondary Metabolite Biosynthesis	CPD-12430	5-guanidino-3-methyl-2-oxopentanoate	-5
Secondary Metabolite Biosynthesis	CPD-824	5-guanidino-2-oxopentanoate	1
Secondary Metabolite Biosynthesis	INDOLE	indole	1
Secondary Metabolite Biosynthesis	CPD-8686	9-oxononanoate	-13
Secondary Metabolite Biosynthesis	CPD-5923	5'-deoxy-5'-fluoroadenosine	-7
Secondary Metabolite Biosynthesis	CPD-18772	gadusol	-11
Secondary Metabolite Biosynthesis	CPD-17453	dithiolgliotoxin	6
Secondary Metabolite Biosynthesis	CPD-17053	bis(methylsulfanyl)gliotoxin	12
Secondary Metabolite Biosynthesis	CPD-17455	mono(methylsulfanyl)gliotoxin	6
Secondary Metabolite Biosynthesis	CPD-17043	cyclo(L-phenylalanyl-L-seryl)	-13
Secondary Metabolite Biosynthesis	CPD-9700	hypoglycin B	-7
Secondary Metabolite Biosynthesis	CPD-9699	hypoglycin A	16
Secondary Metabolite Biosynthesis	CPD-10525	indigo	-14
Secondary Metabolite Biosynthesis	CPD-14625	spinosyn macrolactone	14
Secondary Metabolite Biosynthesis	CPD-14629	spinosyn tricyclic macrolactone	8
Secondary Metabolite Biosynthesis	CPD-13385	spinosyn A	-10
Secondary Metabolite Biosynthesis	CPD-463	L-nicotianamine	-6
Secondary Metabolite Biosynthesis	CPD-18328	lyngbyatoxin A	7
Secondary Metabolite Biosynthesis	BENZOATE	benzoate	8
Secondary Metabolite Biosynthesis	CAPSAICIN	capsaicin	9
Secondary Metabolite Biosynthesis	CPD-9329	8-methyl-6-nonenoate	-13
Secondary Metabolite Biosynthesis	O-UREIDOHOMOSERINE	O-ureido-L-homoserine	15
Secondary Metabolite Biosynthesis	CANAVANINOSUCCINATE	canavaninosuccinate	1
Secondary Metabolite Biosynthesis	L-CANALINE	L-canaline	13
Secondary Metabolite Biosynthesis	BENZYL-DESULFOGLUCOSINOLATE	desulfoglucotropaeolin	13
Secondary Metabolite Biosynthesis	CPD-8001	calystegine A3	-7
Secondary Metabolite Biosynthesis	CPD-17697	tomatidine	-8
Secondary Metabolite Biosynthesis	CPD-18641	tomatidine galactoside	1
Secondary Metabolite Biosynthesis	CPD-8935	feruloylserotonin	-7
Secondary Metabolite Biosynthesis	CPD-8987	senecionine N-oxide	8
Secondary Metabolite Biosynthesis	CPD-1103	taxiphyllin	-14
Secondary Metabolite Biosynthesis	CPD-11852	dehydroscoulerine	-13
Secondary Metabolite Biosynthesis	CPD-8658	amaranthin	-6
Secondary Metabolite Biosynthesis	CPD-17338	peramine	-1
Secondary Metabolite Biosynthesis	CPD-16985	ardeemin	-14
Secondary Metabolite Biosynthesis	INDOLEYL-CPD	(indole-3-yl)acetonitrile	-7
Secondary Metabolite Biosynthesis	2-AMINO-4-CARBOXYPYRIMIDINE	2-amino-4-carboxypyrimidine	1
Secondary Metabolite Biosynthesis	N-EE-PIPEROYL-PIPERIDINE	piperine	-1
Secondary Metabolite Biosynthesis	CPDQT-418	7-(methylsulfanyl)heptyl-desulfoglucosinolate	-6
Secondary Metabolite Biosynthesis	CPD-9217	solanidine	-7
Secondary Metabolite Biosynthesis	CPD-8941	cinnamoyltyramine	-7
Secondary Metabolite Biosynthesis	CPD-8943	p-coumaroyltyramine	-15
Secondary Metabolite Biosynthesis	VINORINE	vinorine	13
Secondary Metabolite Biosynthesis	CPD-14751	ajmaline	-8
Secondary Metabolite Biosynthesis	CPD-4243	sarpagine	14
Secondary Metabolite Biosynthesis	CPD-2748	nornicotine	13
Secondary Metabolite Biosynthesis	CPD-10277	lotaustralin	14
Secondary Metabolite Biosynthesis	CPD-18646	furostanol-26-alcohol	8
Secondary Metabolite Biosynthesis	CPD-19447	11-hydroxyvittatine	-14
Secondary Metabolite Biosynthesis	CPD-19438	lycorine	-8
Secondary Metabolite Biosynthesis	CPD-12600	2-arachidonoylglycerol	-1
Secondary Metabolite Biosynthesis	CPD-446	N-glucosylnicotinate	-13
Secondary Metabolite Biosynthesis	PYRIDOXINE	pyridoxine	-7
Secondary Metabolite Biosynthesis	PYRIDOXAL_PHOSPHATE	pyridoxal 5'-phosphate (PLP)	18
Secondary Metabolite Biosynthesis	CPD-12421	2-benzoyloxy-3-butenylglucosinolate	5
Secondary Metabolite Biosynthesis	CPDQT-274	4-hydroxybutylglucosinolate	17
Secondary Metabolite Biosynthesis	CPD-17022	asperlicin D	-3
Secondary Metabolite Biosynthesis	CPD-17021	asperlicin C	-15
Secondary Metabolite Biosynthesis	CPD-16970	fumiquinazoline A	1
Secondary Metabolite Biosynthesis	CPD-16899	(R)-benzodiazepinedione	1
Secondary Metabolite Biosynthesis	CPD-9936	(S)-magnoflorine	8
Secondary Metabolite Biosynthesis	CPD-17881	fumiquinazoline F-indoline-3'-ol	7
Secondary Metabolite Biosynthesis	CPD-17886	deoxynortryptoquialanone	-2
Secondary Metabolite Biosynthesis	CPD-17888	tryptoquialanol	-12
Secondary Metabolite Biosynthesis	3-URACIL-1-YL-L-SERINE	L-willardiine	-7
Secondary Metabolite Biosynthesis	XANTHOSINE	xanthosine	7
Secondary Metabolite Biosynthesis	7-METHYLXANTHOSINE	7-methylxanthosine	-14
Secondary Metabolite Biosynthesis	7-METHYLXANTHINE	7-methylxanthine	-6
Secondary Metabolite Biosynthesis	CPD-8919	17-oxosparteine	9
Secondary Metabolite Biosynthesis	CPD-18299	4'-methoxycyclopeptine	9
Secondary Metabolite Biosynthesis	XANTHINE	xanthine	-8
Secondary Metabolite Biosynthesis	CPD-15260	fumitremorgin B	-8
Secondary Metabolite Biosynthesis	CPD-15261	fumitremorgin A	-2
Secondary Metabolite Biosynthesis	CPD-12232	hordatine A	15
Secondary Metabolite Biosynthesis	CPD-8757	betaxanthin	2
Secondary Metabolite Biosynthesis	CPDQT-419	8-(methylsulfanyl)octyl-desulfoglucosinolate	9
Secondary Metabolite Biosynthesis	RICININE	ricinine	6
Secondary Metabolite Biosynthesis	CPD-9941	harmol	2
Secondary Metabolite Biosynthesis	6-O-METHYLNORLAUDANOSOLINE	(S)-6-O-methylnorlaudanosoline	-5
Secondary Metabolite Biosynthesis	CPD-8659	miraxanthin V	5
Secondary Metabolite Biosynthesis	CPD-15267	demethoxyfumitremorgin C	2
Secondary Metabolite Biosynthesis	CPD-15262	tryprostatin A	7
Secondary Metabolite Biosynthesis	CPD-17361	notoamide D	7
Secondary Metabolite Biosynthesis	CPDQT-417	6-(methylsulfanyl)hexyl-desulfoglucosinolate	-18
Secondary Metabolite Biosynthesis	CPD-17389	glandicoline A	-7
Secondary Metabolite Biosynthesis	CPD-17390	glandicoline B	7

Secondary Metabolite Biosynthesis	CPD-17393	roquefortine F	7
Secondary Metabolite Biosynthesis	CPD-9788	O-methylandrocymbine	14
Secondary Metabolite Biosynthesis	S-NORLAUDANOSOLINE	(S)-norlaudanosoline	-13
Secondary Metabolite Biosynthesis	17-O-DEACETYLVINDOLINE	17-O-deacetylvindoline	8
Secondary Metabolite Biosynthesis	CPD-7858	vinblastine	-1
Secondary Metabolite Biosynthesis	CPD-9981	7-deoxyloganate	-6
Secondary Metabolite Biosynthesis	LOGANIN	loganin	7
Secondary Metabolite Biosynthesis	PROTOPINE	protopine	-14
Secondary Metabolite Biosynthesis	CPD-9893	chelirubine	7
Secondary Metabolite Biosynthesis	BERBAMUNINE	berbamunine	-22
Secondary Metabolite Biosynthesis	CPD-8851	dopamine quinone	6
Secondary Metabolite Biosynthesis	CPD-14816	cephaeline	8
Secondary Metabolite Biosynthesis	CPD-460	4-(3-methylbut-2-enyl)-L-tryptophan	-14
Secondary Metabolite Biosynthesis	CPD-12352	4-(3-methylbut-2-enyl)-L-abrine	-12
Secondary Metabolite Biosynthesis	CPD-12356	chanoclavine-I	-19
Secondary Metabolite Biosynthesis	CPD-12363	ergotamine	-7
Secondary Metabolite Biosynthesis	CPD-12357	agroclavine	8
Secondary Metabolite Biosynthesis	AMINO-ETCETERA-PYRIDIN-1-YL-PROPANOATE	L-mimosine	20
Secondary Metabolite Biosynthesis	CPD-19480	2-[(2'-methylsulfanyl)hexyl]maleate	1
Secondary Metabolite Biosynthesis	CPDQT-35	2-[(7'-methylsulfanyl)heptyl]malate	-7
Secondary Metabolite Biosynthesis	CPDQT-277	L-hexahomomethionine	14
Secondary Metabolite Biosynthesis	CPDQT-404	L-trihomomethionine	14
Secondary Metabolite Biosynthesis	CPDQT-28	7-(methylsulfanyl)-2-oxoheptanoate	-13
Secondary Metabolite Biosynthesis	CPDQT-29	8-(methylsulfanyl)-2-oxooctanoate	-4
Secondary Metabolite Biosynthesis	CPD-17378	histidyltryptophyldiketopiperazine	-21
Secondary Metabolite Biosynthesis	CPD-15265	brevianamide F	4
Secondary Metabolite Biosynthesis	CPD-7996	littorine	-7
Secondary Metabolite Biosynthesis	SCOPOLAMINE	scopolamine	-22
Secondary Metabolite Biosynthesis	CPD-16844	didemethylasterriquinone D	6
Secondary Metabolite Biosynthesis	CPD-16846	terrequinone A	6
Secondary Metabolite Biosynthesis	CPD-14465	dihydrochanoclavine-I aldehyde	10
Secondary Metabolite Biosynthesis	CPD-14469	fumigaclavine C	-7
Secondary Metabolite Biosynthesis	BERBERINE	berberine	-7
Secondary Metabolite Biosynthesis	CODEINONE	codeinone	-7
Secondary Metabolite Biosynthesis	CODEINE	codeine	-13
Secondary Metabolite Biosynthesis	R-RETICULINE	(R)-reticuline	15
Secondary Metabolite Biosynthesis	DOPAQUINONE	dopaquinone	-6
Secondary Metabolite Biosynthesis	CPD-9835	cinchonidine	-13
Secondary Metabolite Biosynthesis	CPD-9851	corynantheal	-6
Secondary Metabolite Biosynthesis	CPD-9848	quinidinone	-7
Secondary Metabolite Biosynthesis	CPD-9846	quinidine	8
Secondary Metabolite Biosynthesis	3-HYDROXY-L-KYNURENINE	3-hydroxy-L-kynurenine	13
Secondary Metabolite Biosynthesis	LICODIONE	licodione	1
Secondary Metabolite Biosynthesis	CPD-1962	(+)-afzelechin	1
Secondary Metabolite Biosynthesis	CPD-9711	dalcochinin	8
Secondary Metabolite Biosynthesis	CPD-17599	(-)-bursehernin	-8
Secondary Metabolite Biosynthesis	CPD-18755	(-)-5'-demethylyatein	12
Secondary Metabolite Biosynthesis	CPD-9588	maysin	12
Secondary Metabolite Biosynthesis	CPD-14619	atromentin	-2
Secondary Metabolite Biosynthesis	6-METHOXYMELLEIN	6-methoxymellein	13
Secondary Metabolite Biosynthesis	CPD-10256	5-pentadecatrienyl resorcinol	-8
Secondary Metabolite Biosynthesis	CPD-12348	methoxydihydrosorgoleone	-4
Secondary Metabolite Biosynthesis	CPD-10258	dihydrosorgoleone	14
Secondary Metabolite Biosynthesis	CPD-8949	tellimagrandin II	12
Secondary Metabolite Biosynthesis	CPD-7119	xanthohumol	-7
Secondary Metabolite Biosynthesis	CPD-7075	hesperidin	-1
Secondary Metabolite Biosynthesis	CPD-14960	kaempferide triglycoside	12
Secondary Metabolite Biosynthesis	CPD-9318	3-geranyl-4-hydroxybenzoate	19
Secondary Metabolite Biosynthesis	CPD-9320	3''-hydroxy-geranylhydroquinone	10
Secondary Metabolite Biosynthesis	SULOCHRIN	sulochrin	-6
Secondary Metabolite Biosynthesis	CPD-12037	hispidol	-16
Secondary Metabolite Biosynthesis	CPD-14848	genistin 7-gentiobioside	21
Secondary Metabolite Biosynthesis	CPD-6964	pinosylvin monomethylether	14
Secondary Metabolite Biosynthesis	PHENYLACETALDEHYDE	phenylacetaldehyde	-23
Secondary Metabolite Biosynthesis	CPD-9561	protohypericin	14
Secondary Metabolite Biosynthesis	CPD-6602	curcumin	13
Secondary Metabolite Biosynthesis	CPD-9536	5'-hydroxy-rot-2'-enonate	7
Secondary Metabolite Biosynthesis	CPD-7026	afrormosin	13
Secondary Metabolite Biosynthesis	CPD-9534	rot-2'-enonate	8
Secondary Metabolite Biosynthesis	CPD-16749	5'-demethoxy-6-methoxypodophyllotoxin	2
Secondary Metabolite Biosynthesis	CPD-19955	butyl benzoate	-15
Secondary Metabolite Biosynthesis	CPD-18998	propyl acetate	9
Secondary Metabolite Biosynthesis	KIEVITONE-CPD	kievitone	5
Secondary Metabolite Biosynthesis	MYRICETIN	myricetin	-19
Secondary Metabolite Biosynthesis	CPD-11530	dihydroconiferyl aldehyde	-6
Secondary Metabolite Biosynthesis	CPD-17703	coniferyl alcohol 9-methyl ester	1
Secondary Metabolite Biosynthesis	CPD-7252	kaempferide	1
Secondary Metabolite Biosynthesis	CPD-79	sinapaldehyde glucoside	-13
Secondary Metabolite Biosynthesis	CPD-8921	(+)-piperitol	-13
Secondary Metabolite Biosynthesis	CPD-18761	coniferyl alcohol radical	7
Secondary Metabolite Biosynthesis	CPD-13494	6-gingerol	7
Secondary Metabolite Biosynthesis	CPD-8184	chrysin	-16
Secondary Metabolite Biosynthesis	CPD-6991	(2S)-pinocembrin	1
Secondary Metabolite Biosynthesis	CPD-20014	vicenin-2	22
Secondary Metabolite Biosynthesis	PHLORETIN	phloretin	1
Secondary Metabolite Biosynthesis	574-TRIHYDROXY-3-METHOXYFLAVONE	chrysoeriol	-12
Secondary Metabolite Biosynthesis	SINAPATE	sinapate	-12
Secondary Metabolite Biosynthesis	CPD-6481	eugenol	-14
Secondary Metabolite Biosynthesis	CPD-4885	(-)-phaseollidin	-1
Secondary Metabolite Biosynthesis	CPD-13543	kauralexin B2	7
Secondary Metabolite Biosynthesis	CPD-13544	kauralexin B3	-8
Secondary Metabolite Biosynthesis	INDOLE-CARBOXY-HYDRO-THIAZOLE	(R)-dihydrocamalexate	1
Secondary Metabolite Biosynthesis	CPD-4745	rishitin	-1
Secondary Metabolite Biosynthesis	CPD-4746	3-hydroxy-15-dihydrolubimin	8
Secondary Metabolite Biosynthesis	CPD-18574	3-hydroxy-5-methoxybiphenyl	-2
Secondary Metabolite Biosynthesis	CPD-4461	plumbagin	-15
Secondary Metabolite Biosynthesis	CPD-12673	5-chloro-5-deoxy-D-ribonate	8
Secondary Metabolite Biosynthesis	CPD-11020	5-chloro-4-hydroxy-2-oxopentanoate	2
Secondary Metabolite Biosynthesis	CPD-12679	4-chloro-3-hydroxybutyryl-CoA	-14

Secondary Metabolite Biosynthesis	CPD-12680	4-chloro-crotonyl-CoA	6
Secondary Metabolite Biosynthesis	CPD-12684	L-3-cyclohex-2'-enylalanine	-1
Secondary Metabolite Biosynthesis	CPD-12201	anigorufone	1
Secondary Metabolite Biosynthesis	CPD-18211	6-hydroxy-norbikaverin	-16
Secondary Metabolite Biosynthesis	CPD-18032	fusarinolate	6
Secondary Metabolite Biosynthesis	CPD-18034	methyl fusarate	1
Secondary Metabolite Biosynthesis	CPD-18031	fusarate	13
Secondary Metabolite Biosynthesis	CPD-10679	flaviolin	-16
Secondary Metabolite Biosynthesis	CPD-11587	chrysophanol anthrone	-6
Secondary Metabolite Biosynthesis	CPD-18255	YWA1	-18
Secondary Metabolite Biosynthesis	CPD-18244	dimeric 9-hydroxyrubrofusarin	12
Secondary Metabolite Biosynthesis	CPD-6801	aloesone	-2
Secondary Metabolite Biosynthesis	CPD-12180	feruloylacetyl-CoA	15
Secondary Metabolite Biosynthesis	CPD-17133	desmethylbassianin	-2
Secondary Metabolite Biosynthesis	CPD-17131	predesmethylbassianin A	8
Secondary Metabolite Biosynthesis	CPD-20577	norbaeocystin	7
Secondary Metabolite Biosynthesis	CPD-20579	baeocystin	13
Secondary Metabolite Biosynthesis	CPD-20104	dihydrorhizobitoxine	-16
Secondary Metabolite Biosynthesis	CPD-18452	cylindrospermopsin	13
Secondary Metabolite Biosynthesis	CPD-21113	felinine	6
Secondary Metabolite Biosynthesis	L-CYSTEATE	L-cysteate	12
Secondary Metabolite Biosynthesis	CPD-12491	phenylmethanesulfenate	-7
Secondary Metabolite Biosynthesis	2-AMINOACRYLATE	2-aminoprop-2-enoate	-17
Secondary Metabolite Biosynthesis	CPD-18776	(R)-4-deoxygadusol	5
Secondary Metabolite Biosynthesis	CPD-3764	desferrioxamine B	8
Secondary Metabolite Biosynthesis	CPD0-2241	ferrichrome	-16
Secondary Metabolite Biosynthesis	CPD-9990	anguibactin	8
Secondary Metabolite Biosynthesis	CPD-11965	pre-putrebactin	-4
Secondary Metabolite Biosynthesis	CPD-9985	petrobactin	11
Secondary Metabolite Biosynthesis	CPD-11968	alcaligin	8
Secondary Metabolite Biosynthesis	CPD-7836	myristate	13
Secondary Metabolite Biosynthesis	CPD-12313	schizokinen	-7
Secondary Metabolite Biosynthesis	CPD-20999	pseudomonine	-22
Secondary Metabolite Biosynthesis	CPD-21000	pre-pseudomonine	-2
Secondary Metabolite Biosynthesis	CPD-21009	baumannoferrin A	16
Secondary Metabolite Biosynthesis	CPD-7228	3''-deamino-3''-oxonicotianamine	-7
Secondary Metabolite Biosynthesis	CPD-11970	bisucaberin	-7
Secondary Metabolite Biosynthesis	CPD-9986	achromobactin	6
Secondary Metabolite Biosynthesis	CPD-7289	3-epihydroxymugineate	-14
Secondary Metabolite Biosynthesis	CPD-7291	3-epihydroxy-2'-deoxymugineate	7
Secondary Metabolite Biosynthesis	CPD-12151	D-histidine	7
Secondary Metabolite Biosynthesis	CPD-10176	versicolorin A	6
Secondary Metabolite Biosynthesis	CPD-12930	neurosporaxanthin	-7
Secondary Metabolite Biosynthesis	CPD-9481	hederagenin	1
Secondary Metabolite Biosynthesis	CPD-9482	soyasapogenol E	1
Secondary Metabolite Biosynthesis	CPD-7968	bixin aldehyde	14
Secondary Metabolite Biosynthesis	CPD-7972	bixin	9
Secondary Metabolite Biosynthesis	CPD-8662	crocetin	-19
Secondary Metabolite Biosynthesis	CPD-8668	picrocrocin	7
Secondary Metabolite Biosynthesis	CPD-14499	erythrodiol	-8
Secondary Metabolite Biosynthesis	CPD-20690	fucoxanthinol	1
Secondary Metabolite Biosynthesis	CPD-14485	glycyrrhetaldehyde	16
Secondary Metabolite Biosynthesis	CPD-18415	3-acetyl T-2 toxin	12
Secondary Metabolite Biosynthesis	CPD-10662	C30 botryococcene	-12
Secondary Metabolite Biosynthesis	CPD-14289	C33 botryococcene	-15
Secondary Metabolite Biosynthesis	CPD-9528	desoxyhemigossypol-6-methyl ether	-13
Secondary Metabolite Biosynthesis	CPD-17204	lolitriol	7
Secondary Metabolite Biosynthesis	CPD-17196	terpendole I	-8
Secondary Metabolite Biosynthesis	CPD-16667	cyclooctat-9-en-7-ol	7
Secondary Metabolite Biosynthesis	CPD-17660	2-hydroxycanthaxanthin	9
Secondary Metabolite Biosynthesis	CPD-17662	2-hydroxyadonixanthin	-16
Secondary Metabolite Biosynthesis	CPD-14505	rubusoside	-15
Secondary Metabolite Biosynthesis	CPD-7003	tetrahydrogeranylgeranyl diphosphate	4
Secondary Metabolite Biosynthesis	CPD-7002	dihydrogeranylgeranyl diphosphate	7
Secondary Metabolite Biosynthesis	CPD-17491	astaxanthin dirhamnoside	5
Secondary Metabolite Biosynthesis	CPD-14519	bacteriohopanetetrol	8
Secondary Metabolite Biosynthesis	CPD-14521	aminobacteriohopanetriol	8
Secondary Metabolite Biosynthesis	CPD-14517	2-methyltetrahymanol	-7
Secondary Metabolite Biosynthesis	CPD-18508	hydroxysqualene	8
Secondary Metabolite Biosynthesis	CPD-17304	okenone	-1
Secondary Metabolite Biosynthesis	CPD-9525	hemigossypol	7
Secondary Metabolite Biosynthesis	CPD-9744	heliocide H4	-7
Secondary Metabolite Biosynthesis	BACCATIN-III	baccatin III	-7
Secondary Metabolite Biosynthesis	10-DEACETYL-2-DEBENZOYLBACCATIN-III	10-deacetyl-2-debenzoylbaccatin III	-8
Secondary Metabolite Biosynthesis	CPD-11442	botrydial	-13
Secondary Metabolite Biosynthesis	CPD-18554	stellatic alcohol	8
Secondary Metabolite Biosynthesis	CPD-16861	2-hydroxyspirilloxanthin	8
Secondary Metabolite Biosynthesis	CPD-12615	spirilloxanthin	-8
Secondary Metabolite Biosynthesis	CPD-9758	geranyl acetate	-1
Secondary Metabolite Biosynthesis	CPD-7559	dihydroartemisinate	-7
Secondary Metabolite Biosynthesis	CPD-18410	15-decalonectrin	-21
Secondary Metabolite Biosynthesis	CPD-464	prephytoene diphosphate	-7
Secondary Metabolite Biosynthesis	CPD-18005	dihydroisopentenyldehydrorhodopin	-9
Secondary Metabolite Biosynthesis	CPD-18004	bacterioruberin	-1
Secondary Metabolite Biosynthesis	CPD-18021	monoanhydrobacterioruberin	1
Secondary Metabolite Biosynthesis	CPD-20624	isorenieratene	-8
Secondary Metabolite Biosynthesis	CPD-11469	methoxyneurosporene	-17
Secondary Metabolite Biosynthesis	CPD-11464	demethylspheroidene	8
Secondary Metabolite Biosynthesis	CPD-8723	dehydroabietadiene-diol	-8
Secondary Metabolite Biosynthesis	CPD-8722	dehydroabietadienol	15
Secondary Metabolite Biosynthesis	CPD-8721	abietatriene	6
Secondary Metabolite Biosynthesis	CPD-10307	arabidiol	16
Secondary Metabolite Biosynthesis	CPD-9475	gypsogenate-28-beta-D-glucoside	8
Secondary Metabolite Biosynthesis	CPD-9474	gypsogenate	8
Secondary Metabolite Biosynthesis	CPD-14495	ursolic aldehyde	-15
Secondary Metabolite Biosynthesis	ALL-TRANS-PENTAPRENYL-DIPHOSPHATE	geranylfarnesyl diphosphate (GFPP)	7
Secondary Metabolite Biosynthesis	CPD-707	campesterol	-21
Secondary Metabolite Biosynthesis	CPD-7850	echinenone	-15
Secondary Metabolite Biosynthesis	CPD-16623	des-acyl avenacin A	6

Secondary Metabolite Biosynthesis	CPD-11505	olivetol	8
Secondary Metabolite Biosynthesis	CPD-9635	hyperforin	1
Secondary Metabolite Biosynthesis	CPD-7110	lupulone	-22
Secondary Metabolite Biosynthesis	CPD-19790	adlupulone	2
Secondary Metabolite Biosynthesis	CPD-1086	5-amino-6-(5-phospho-D-ribitylamino)uracil	19
Secondary Metabolite Biosynthesis	AMINO-RIBOSYLAMINO-1H-3H-PYR-DIONE	5-amino-6-(D-ribitylamino)uracil (ARP)	-7
Secondary Metabolite Biosynthesis	CPD-10171	versiconal	-6
Secondary Metabolite Biosynthesis	CPD-14318	deoxyviolacein	7
Secondary Metabolite Biosynthesis	CPD-14323	deoxyviolaceinate	6
Secondary Metabolite Biosynthesis	CPD-14322	prodeoxyviolacein	-2
Secondary Metabolite Biosynthesis	CPD-14319	protoviolaceinate	13
Secondary Metabolite Biosynthesis	CPD-264	3-hydroxybenzoyl-CoA	8
Cell Structure Biosynthesis	GLYCEROL	glycerol	5
Cell Structure Biosynthesis	UMP	UMP	6
Cell Structure Biosynthesis	TDP	dTDP	-5
Cell Structure Biosynthesis	CPD-11591	16-feruloyloxypalmitate	1
Cell Structure Biosynthesis	DODECANOATE	laurate	8
Cell Structure Biosynthesis	CPD-17645	2-(8-hydroxy-2-oxotridecyl)-6-oxopyran-4-olate	18
Cell Structure Biosynthesis	CPD-10512	16-oxo-palmitate	-6
Cell Structure Biosynthesis	CPD-12247	D-alanyl-D-lactate	-17
Metabolic Regulator Biosynthesis	CPD-10470	dimethylsulfonio-2-hydroxybutanoate	-6
Metabolic Regulator Biosynthesis	MET	L-methionine	7
Metabolic Regulator Biosynthesis	CPD-16905	monacolin L acid	4
Metabolic Regulator Biosynthesis	CPD-16924	lovastatin acid	7
Metabolic Regulator Biosynthesis	ACETYL-COA	acetyl-CoA	-6
Metabolic Regulator Biosynthesis	GTP	GTP	-22
Metabolic Regulator Biosynthesis	DPG	3-phospho-D-glyceroyl phosphate (13-DPG)	13
Other Biosynthesis	ACETYL-COA	acetyl-CoA	-6
Other Biosynthesis	CPD-10793	hydroxypyruvaldehyde phosphate	2
Other Biosynthesis	CPD-10791	6-deoxy-5-ketofructose 1-phosphate	7
Other Biosynthesis	CPD-15467	3-dimethylallyl-4-hydroxyphenylpyruvate	-12
Other Biosynthesis	CPD-10600	3-(4-hydroxyphenyl)-3-oxo-propanoyl-CoA	9
Other Biosynthesis	TYR	L-tyrosine	14
Other Biosynthesis	CPD-205	pimelate	-13
Other Biosynthesis	BENZOATE	benzoate	8
Other Biosynthesis	SHIKIMATE-5P	shikimate 3-phosphate	-11
Other Biosynthesis	DIPHTHAMIDE	a diphthamide-[translation elongation factor 2]	-16
Other Biosynthesis	GTP	GTP	-22
Other Biosynthesis	CPD-16457	aurodrosopterin	13
Other Biosynthesis	DOPAQUINONE	dopaquinone	-6
Other Biosynthesis	MET	L-methionine	7
Other Biosynthesis	CPD-20340	ophthalmate	-20
Other Biosynthesis	NAPHTHOL	1-naphthol	-6
Other Biosynthesis	CYS	L-cysteine	13
Other Biosynthesis	CPD-20554	pentabromopseudilin	-9
Other Biosynthesis	CPD-20571	(L-prolyl)adenylate	-6
Other Biosynthesis	2-AMINOACRYLATE	2-aminoprop-2-enoate	-17
Other Biosynthesis	SELENOHOMOCYSTEINE	seleno-L-homocysteine	11
Other Biosynthesis	CPD-20564	spongiadioxin C	7
Other Biosynthesis	TRP	L-tryptophan (trp)	12
